# Ginger and Its Purified Major Components Inhibit Clinically Relevant Uptake and Efflux Transporters In Vitro

**DOI:** 10.3390/pharmaceutics18020149

**Published:** 2026-01-23

**Authors:** Tamás Varga, Nóra Szilvásy, Zsuzsanna Schelz, Renáta Kanizsainé Minorics, Katalin Veres, Csilla Temesszentandrási-Ambrus, Péter Tátrai, Judit Hohmann, Zsuzsanna Gáborik, Emese Kis

**Affiliations:** 1 Doctoral School of Biology, ELTE Eötvös Loránd University, H-1117 Budapest, Hungary; tamas.varga@crl.com; 2 Charles River Laboratories Hungary, H-1117 Budapest, Hungary; nora.szilvasy@crl.com (N.S.); csilla.temesszentandrasi-ambrus@crl.com (C.T.-A.); peter.tatrai@crl.com (P.T.); zsuzsanna.gaborik@crl.com (Z.G.); 3 Institute of Pharmacodynamics and Biopharmacy, University of Szeged, H-6720 Szeged, Hungary; schelz.zsuzsanna@szte.hu (Z.S.); kanizsaine.minorics.renata@szte.hu (R.K.M.); 4 Institute of Pharmacognosy, University of Szeged, H-6720 Szeged, Hungary; veres.katalin@szte.hu (K.V.); hohmann.judit@szte.hu (J.H.); 5 HUN-REN–SZTE Biologically Active Natural Products Research Group, University of Szeged, H-6720 Szeged, Hungary

**Keywords:** *Zingiber officinale*, gingerol, shogaol, solute carrier proteins, ATP-binding cassette transporters, herb–drug interaction

## Abstract

**Background/Objectives**: Ginger (*Zingiber officinale* Roscoe) is a flowering plant widely used as a spice and natural medicine for millennia. Ginger demonstrates multiple protective effects, regulates cholesterol, and may reduce the risk of cancer and colitis. However, little attention has been paid to its potential to cause herb–drug interactions (HDIs). The aim of this study was to investigate the interaction of ginger extract and its major components [6]-gingerol and [6]-shogaol with clinically relevant uptake and efflux transporters in vitro. **Methods**: Transporter-overexpressing cell lines of 25 uptake transporters and inside-out membrane vesicles containing 8 efflux transporters were employed to measure potential interactions. **Results**: *Zingiber officinale* extract at 150 µg/mL interacted with 17 of 33 transporters examined. These were further investigated for interactions with the purified active components. Seven and 16 transporters interacted with pure [6]-gingerol (100 µM) and [6]-shogaol (100 µM), respectively. To evaluate the risk of in vivo inhibition, IC_50_ values were determined for the affected transporters. Based on standard risk assessment calculations, we confirmed previously reported inhibitory effects of ginger components on MDR1 (67.64 µM) and BCRP (9.931 µM), and revealed novel potential interactions with renal OAT3 (0.956 µM) and URAT1 (5.887 µM), hepatic OCT1 (4.287 µM) and BSEP (25.45 µM), and the ubiquitously expressed ENT1 (11.62 µM) ([6]-shogaol IC_50_ values are shown in parentheses). Strong and isoform-selective inhibition of OAT3 by [6]-shogaol is particularly intriguing. Additionally, via cell viability experiments on a set of human cervical, breast, and oropharyngeal cancer cell lines, we demonstrated the antiproliferative effect of [6]-shogaol in vitro. **Conclusions**: Prolonged consumption of high-dose ginger supplements may pose a risk of transporter-mediated HDIs when consumed concomitantly with conventional medications. Our study encourages follow-up of the suspected effects in vivo.

## 1. Introduction

Ginger (*Zingiber officinale* Roscoe, Zingiberaceae), a perennial plant native to tropical Asia, is now cultivated globally and used worldwide as a spice and a medicinal plant [1]. For centuries, its rhizome has been a key traditional herbal medicine for the treatment of a variety of conditions such as colds, arthritis, nervous diseases, nausea, gastrointestinal issues, gingivitis, toothache, asthma, stroke, constipation, and diabetes [2,3,4]. Many of these beneficial health effects have been confirmed in systematic studies, including clinical trials. Ginger and its main active components have been shown to exert anticancer [5,6], anti-inflammatory, antioxidant, and antimicrobial activities; reduce body weight and fasting glucose levels in obese individuals [7,8]; alleviate pain [9]; and suppress nausea and vomiting [10,11]. These validated benefits make ginger a vastly popular food supplement, and the ginger market is projected to grow from $4.41 billion in 2024 to $7.50 billion in 2033 [12].

The composition of ginger varies widely depending on the region of cultivation and whether the rhizomes are fresh or dried [13]. The characteristic odor of ginger is primarily attributed to its volatile oil (1–3%), which contains monocyclic sesquiterpenes, with bisabolene and zingiberene as the major compounds [1]. The spiciness of fresh ginger is mainly due to the diarylheptanoid-type gingerols, with [6]-gingerol being the most prevalent compound in this homologous series [14]. Gingerols are among the most studied phytochemicals in ginger, known for their role in alleviating nausea, arthritis, and pain. Some of these gingerols also play a specific role in the treatment of diabetes and various types of tumors [1,8]. The pungency of dry ginger mainly comes from shogaols, such as [6]-shogaol, which are the dehydrated forms of gingerols. Shogaols are formed from the corresponding gingerol during thermal processing. The homologous series of gingerols and shogaols includes 4-, 6-, 7-, 8-, and 10-gingerol, and 4-, 6-, 8-, 10-, and 12-shogaol. Additionally, methyl and isoderivatives, 5-deoxygingerols (paradols), and gingerdiones have also been identified in ginger rhizomes [2,9]. [6]-shogaol exhibits significant anticancer, antioxidant, and anti-inflammatory properties, and is more effective than [6]-gingerol due to the presence of an electrophilic Michael acceptor group [9,15,16].

Despite the widespread use of ginger, the extent to which its bioactive components modulate the disposition of endogenous and exogenous compounds in the human body remains poorly understood. Of interest to our study, herbal components, just like drugs, can interfere with the function of drug transporters expressed at key biological barriers [17]. Drug transporters, categorized into the SLC (solute carrier) and ABC (ATP-binding cassette) superfamilies, are well known for mediating drug–drug interactions (DDIs) [18]. Regulatory agencies have established guidelines for preclinical in vitro transporter studies to be conducted as part of drug development (for the most recent guidance document, see [19]). While comprehensive knowledge has been gathered about the most important transporters and their interactions with commercially available drugs [20], pharmacokinetic research on less tightly regulated herbal supplements is lagging, and transporter-mediated herb–drug interactions have so far received relatively little attention.

The outcome of a transporter-mediated interaction depends on the barrier(s) affected, the nature of the substrate compound, and the direction of transport. The intestinal epithelium expresses a broad range of SLC and ABC transporters that mediate the uptake and efflux of nutrients and xenobiotics [21]. Uptake transporters facilitate the entry of drugs across the apical membrane, while efflux transporters such as multidrug resistance 1 (MDR1), breast cancer resistance protein (BCRP), and multidrug resistance-associated protein 2 (MRP2), limit absorption by pumping drugs back to the intestinal lumen [18]. This bidirectional transporter activity influences oral bioavailability and, when altered, may contribute to DDIs. In the liver, transporter-mediated clearance is critical and often rate-limiting to drug disposition. Uptake transporters located on the sinusoidal side of hepatocytes, such as the organic anion transporting polypeptides OATP1B1 and OATP1B3, facilitate the hepatic entry of drugs and endogenous compounds [22]. Efflux transporters on the canalicular side, including MDR1, BCRP, and MRPs, mediate the excretion of drugs and their metabolites into bile [18]. The interplay between uptake and efflux transporters significantly influences hepatic drug concentration, bioavailability, and potential DDIs. Disruption or inhibition of these transporters can alter pharmacokinetics and toxicity [23]. In the kidney, uptake transporters on the basolateral membrane, such as organic anion transporters (OAT1 and 3), and organic cation transporter 2 (OCT2), mediate the entry of drugs from blood into proximal tubule cells. Efflux across the apical membrane into the tubular lumen is primarily facilitated by transporters such as MDR1, BCRP, MRPs, and multidrug and toxin extrusion proteins (MATE) 1 and 2K. These coordinated actions determine renal clearance, drug half-life, and potential nephrotoxicity. Dysregulation or inhibition of these transporters can significantly impact pharmacokinetics and precipitate DDIs [24].

Enzyme- and transporter-based ginger–drug interactions have been documented in the literature. Ginger extract and its arylheptanoids have been shown to significantly inhibit multiple CYP isoforms, as well as efflux transport via MDR1 and BCRP [25,26,27]. Another study found a synergistic effect on platelet aggregation when ginger was co-administered with nifedipine [28]. Thus, we hypothesize that excessive ginger intake may interfere with drug disposition and increase the risk of herb–drug interactions when ginger is co-administered with conventional medications. In this study, we examined the inhibitory effect of ginger extract and its active components on a selected panel of clinically relevant uptake and efflux transporters in vitro (Table S1) and evaluated the anticipated in vivo relevance of the measured inhibitions. Additionally, to assess potential synergy or excess toxicity of ginger intake concomitant with anticancer therapy, we tested the in vitro antiproliferative effect of [6]-gingerol and [6]-shogaol in a set of human cervical, breast, and oropharyngeal cancer cell lines.

## 2. Materials and Methods

### 2.1. Preparation of Zingiber officinale Extracts

*Chemicals and Reagents*. Analytical-grade methanol (Molar Chemicals, Halásztelek, Hungary) was used for plant extraction. Purified water was obtained using a Millipore Direct-QVR 3 UV pump (Millipore S.A.S., Molsheim, France). HPLC-grade acetonitrile and methanol (MeOH) were used for sample preparation, and HPLC experiments were purchased from Merck (Darmstadt, Germany). [6]-gingerol (Product no. 89201) and [6]-shogaol (Product no. 89792) were acquired from PhytoLab (Vestenbergsgreuth, Germany). All other compounds were obtained from Merck.

*Plant Material*. Fresh ginger rhizomes were purchased from a commercial source in Szeged, Hungary. A small part of the rhizome was preserved as a voucher specimen (No. 945) in a freezer at −20 °C at the Institute of Pharmacognosy, Szeged, Hungary. A total of 274.3 g of fresh *Z. officinale* rhizomes were chopped into 2–4 mm pieces and extracted by percolation using 9 × 500 mL of 90% MeOH. The extract was then filtered and concentrated under vacuum using a Rotavapor instrument until it reached a *spissum* consistency. Subsequently, the MeOH-free extract was dried by lyophilization with a Christ Alpha 1-4 LSCplus instrument (Osterode am Harz, Germany). The yield of the dried extract was 2.95% (8.1 g).

### 2.2. Quantitative Analysis of the Extract

*Instrumentation*. The liquid chromatographic analysis of ginger extract was conducted using a Shimadzu (Kyoto, Japan) system, which included an LC-20 AD pump, a DGU-20ASR degasser unit, an SPD-M20A diode array detector, a CBM-20A controller, a CTO-20AC column oven, and a SIL-20AHT autosampler. Data processing was carried out using LabSolutions software (Version 5.82, Shimadzu, Kyoto, Japan). Chromatographic separation was performed on a Kinetex C-8 column (100 Å, 150 × 4.60 mm, 5 µm, Phenomenex, Torrance, CA, USA) following a gradient program that modified the MeOH content in water (with 0.1% phosphoric acid) as follows: 58% (0–1 min), increasing from 58% to 67% (1–10 min), maintaining 67% for 1 min, rising to 100% (11–12 min), and then decreasing to 60% over 3 min. The flow rate was set to 1.5 mL/min. The analyte was monitored within the UV-Vis range (190–800 nm), with a specific UV_max_ detection at 230 nm for the standards ([6]-gingerol and [6]-shogaol). The column temperature was maintained at 25 °C.

*Validation*. The major components of ginger rhizome, [6]-gingerol and [6]-shogaol, were used as standards to analyze the composition of the extract. The retention time of [6]-gingerol was 3.9 min, while that of [6]-shogaol was 7.9 min (Figure S1). Limit of detection (LoD) values were determined ([6]-gingerol: 1.99 ng/inj; [6]-shogaol: 1.07 ng/inj), and the limit of quantification (LoQ) values were calculated for the standards ([6]-gingerol: 3.23 ng/inj; [6]-shogaol: 6.02 ng/inj). Calibration curves were established based on 8 calibration points (Figure S2). The correlation coefficients of the calibration curves were R^2^ = 0.99879 for [6]-gingerol and R^2^ = 0.99995 for [6]-shogaol.

*Sample preparation*. 125 mg of lyophilized 90% MeOH extract of ginger rhizome was measured into a 25 mL volumetric flask and dissolved in MeOH, aided by an ultrasonic bath. After homogenization, the sample was filtered by a PTFE 0.45 μm filter (FilterBio PTFE-L Syringe filter, Labex, Budapest, Hungary), and the first 0.5 mL was discarded. Three such sample preparations were made from the 90% MeOH extract, and each sample was injected in triplicate. The injection volume was 20 μL. All extracts were stored in the refrigerator until analysis. The composition of the dried extract prepared from fresh rhizomes of ginger with 90% MeOH was characterized by the HPLC-DAD method. The [6]-gingerol content was determined as 24.555 ± 0.6724 mg/g extract (2.455%), and the [6]-shogaol content as 5.861 ± 0.072 mg/g extract (0.586%).

### 2.3. Cell Line Generation, Cell Culturing, and Membrane Preparation

*Generation of transporter-expressing cell lines*. The human embryonic kidney 293 (HEK293) cell line was purchased from Invitrogen/ThermoFisher (Waltham, MA, USA). Madin-Darby canine kidney II (MDCKII) wild-type cells were obtained from the European Collection of Authenticated Cell Cultures (ECACC catalog no. 00062107). Transporter-expressing cell lines were developed by lentiviral transduction of HEK293 or MDCKII cells, utilizing lentiviral reagents from System Biosciences (Palo Alto, CA, USA). Expression cassettes were constructed by GenScript (Piscataway, NJ, USA). Apart from the gene of interest, expression vectors also contained an antibiotic (puromycin, blasticidin, or hygromycin) resistance gene to allow for positive selection of transporter-containing cells. Upon confirmation of successful gene delivery, single-cell colonies of each cell line were created, and selected monoclones were functionally tested for 4 to 6 weeks to ensure stable high performance. Production of lentivirus supernatants, transduction of the cells, cloning, functional characterization, and validation were performed by Charles River Laboratories Hungary Kft. (Budapest, Hungary).

*Cell culturing*. HEK293 cell lines were maintained in Dulbecco’s Modified Eagle Medium (DMEM) supplemented with 1% penicillin/streptomycin, 10% fetal bovine serum (FBS), and 3 µg/mL puromycin or 10–15 µg/mL blasticidin. The cells were kept in a humidified incubator at 37 °C and 5% CO_2_. Cells were passaged twice a week at 80–90% confluency. MDCKII cells were cultured similarly, except that the cells were subcultured three times a week at a seeding density of 4500–9000 cells/cm^2^. For the MDCKII cell lines overexpressing MATE1 and MATE2K, hygromycin was used as a selection marker at a concentration of 100 µg/mL.

*Membrane vesicle preparation*. For efflux transporter-expressing HEK293 cell lines, the presence and quantity of transporters were determined by either Western blotting or functional testing. Membrane vesicles were prepared by Charles River Laboratories Hungary Kft. as per the method published by Sarkadi et al. [29].

### 2.4. Transporter Inhibition Assays

*Compounds*. Reagents and non-radiolabeled chemicals were purchased from Merck. All chemicals were analytical grade. Tritium-labeled estrone-3-sulfate (^3^H-E3S) and N-methyl-quinidine (^3^H-NMQ) were purchased from Biological Research Centre (Szeged, Hungary). The tritiated cholecystokinin octapeptide (^3^H-CCK-8), 1-methyl-4-phenylpyridinium (^3^H-MPP^+^), dehydroepiandrosterone sulfate (^3^H-DHEAS), estradiol 17β-D-glucuronide (^3^H-E_2_17βG), taurocholic acid (^3^H-TC), ^3^H-L-serine, and ^3^H-L-leucine, as well as Ultima Gold XR scintillation fluid, were purchased from PerkinElmer (Waltham, MA, USA). ^14^C-metformin, ^14^C-methyl α-D-glucopyranoside (^14^C-AMG), and ^3^H-tetraethylammonium (^3^H-TEA) were from American Radiolabeled Chemicals (St. Louis, MO, USA). ^3^H-tenofovir, ^3^H-uric acid, ^3^H-adenosine, and ^3^H-uridine were from Moravek Inc. (Brea, CA, USA). Hanks’ Balanced Salt Solution (HBSS) (10×) was purchased from ThermoFisher Scientific (Waltham, MA, USA).

*Uptake assays*. For uptake inhibition assays, uptake transporter (SLC)-expressing cell lines, as well as corresponding mock-transduced cells for background assessment, were seeded in triplicate for each experimental condition at a density of 10^5^ cells per well in 200 µL culturing medium on a poly-D-lysine-coated 96-well plate. After 16–24 h of incubation, cells were washed twice with warm assay buffer (1× HBSS, or Krebs–Henseleit (KH) buffer). After 15 min of preincubation with 50 µL per well of the test article (herbal extract, [6]-gingerol or [6]-shogaol) solution, the preincubation mix was aspirated, and the same test article was added once again, this time combined with the radiolabeled probe substrate of the transporter. A solvent control was included in each experiment, and the total organic solvent concentration was 1.0% *v*/*v* in all conditions. Assay details such as buffer, pH, temperature, duration, and substrate are listed in Supplementary Table S2. At the end of incubation, cells were washed twice with ice-cold buffer and lysed with 50 µL 0.1 M NaOH for 2 min. 35 µL cell lysates were transferred to a scintillation plate, complemented with 150 µL scintillation cocktail per well, and then the plates were analyzed in a MicroBeta^2^ Microplate Counter (PerkinElmer).

*Vesicular transport assays*. Vesicular transport inhibition assays with efflux transporter (BCRP, BSEP, MRP1, MRP2, MRP3, MRP4, MRP5, and MDR1) containing membranes were conducted in suspension using the previously validated parameters shown in Supplementary Table S3. First, the test article (ginger extract or its components) and the probe substrate were dissolved in 50 µL membrane suspension on ice, then the mixture was equilibrated at 32 or 37 °C for 15 min on a flat-bottom 96-well plate. The assay was started by adding 25 µL prewarmed ATP (signal group) or AMP (background group) to reach a final volume of 75 µL and a final ATP or AMP concentration of 4 mM. A solvent control was included in each experiment, and the total organic solvent concentration was 1.0% *v*/*v* in all conditions. The assay was stopped by adding 200 µL of ice-cold stop buffer. Well contents were transferred to a MultiScreenHTS-FB filter plate (MSFBN6B50, Millipore), and liquid was removed by vacuum suctioning. Membrane vesicles atop the filters were washed five more times with stop buffer, then the plate was dried at 40 °C in a laboratory oven. Scintillation cocktail was dispensed to the wells once filters were fully dried, and the plate was read in a MicroBeta2 Microplate Counter.

**Figure 1:**
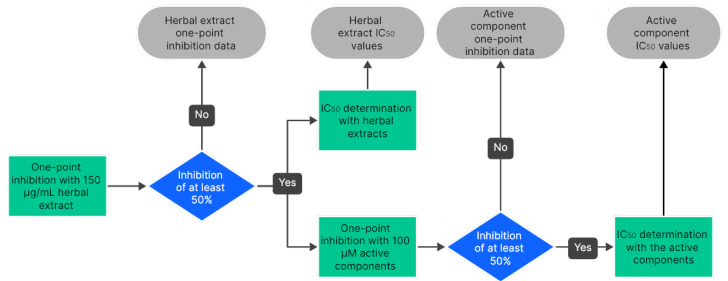
Workflow of the measurements with *Zingiber officinale* extract and its active components, [6]-gingerol and [6]-shogaol.

*Workflow*. A 15 mg/mL stock solution was prepared from *Zingiber officinale* extract, which was diluted in the assay 100-fold to a final concentration of 150 µg/mL. If inhibition at 150 µg/mL exceeded 50% relative to the solvent control, IC_50_ values were determined in independent experiments starting from an extract concentration of 150 µg/mL, and 1-point inhibition studies also took place with 100 µM of the active herbal components ([6]-shogaol and [6]-gingerol). Additionally, all the transporters with regulatory recommendations in the ICH M12 guidance (MDR1, BCRP, OATP1B1, OATP1B3, OAT1, OAT3, OCT2, MATE1, and MATE2K) [19] were also analyzed with the active components. For transporters inhibited by at least 50% by [6]-shogaol and/or [6]-gingerol, IC_50_ values were determined with the respective active components, too. A schematic overview of the workflow is shown in Figure 1. The risk of clinically relevant interactions was evaluated based on available data about in vivo concentrations of ginger ingredients in different body fluids.

### 2.5. Calculations

For one-point inhibition assays, the transporter-specific uptake was assessed by deducting the signal measured in the control cells from the signal measured in the transporter-overexpressing cells. Similarly, for the vesicular transport assays, transporter-specific efflux was assessed by deducting the signal measured in the presence of AMP from the signal measured in the presence of ATP. The calculated specific accumulation was quantified relative to the solvent (DMSO) control and expressed as a percentage. The data represent the mean ± SEM of three replicates. For IC_50_ calculations, dose–response relationships were modeled using a four-parameter nonlinear regression in GraphPad Prism (GraphPad, San Diego, CA, USA).

### 2.6. Risk Assessment

To obtain a comprehensive risk assessment of the observed inhibitions, intestinal, renal, and hepatic in vivo concentrations were compared to the measured IC_50_ values. For the transporters with instructions in the ICH M12 guidance [19], the corresponding cutoff value was also applied in the evaluation process. Of note, in vivo concentrations of ginger components are inconsistent across different sources, which makes interpretation challenging. Zick et al. [30] and Yu et al. [31] both measured the plasma concentrations of [6]-gingerol, [8]-gingerol, [10]-gingerol, and [6]-shogaol in glucuronated, sulfated, and unconjugated forms after a single oral dose of 2000 mg ginger extract, while Levita et al. [32] determined the plasma concentrations of the unconjugated forms of [10]-gingerol and [6]-shogaol after the same dosage. A summary of the data for [6]-gingerol and [6]-shogaol is shown in Table 1.

In our analysis, renal and hepatic transporter calculations were based on the highest available C_max_ values (marked in bold in Table 1), which translate to 2.89 µM for [6]-gingerol and 1.64 µM for [6]-shogaol. Although the unconjugated form of [6]-gingerol was not detected in the serum of healthy humans, the C_max_ (total) may still be relevant as a theoretical maximum of free [6]-gingerol serum concentration. In another study by Schoenknecht et al. [33], the unconjugated form of [6]-gingerol was detectable in human plasma (C_max_(uncon) = 0.042 µM), but this concentration was quantified after ginger tea consumption. Ginger tea generally contains lower concentrations of active constituents compared with ginger extracts. In a study by Schwertner et al., the analyzed extract (Enzymatic Therapy^®^, Green Bay, WI, USA) contained 4.78% [6]-gingerol and 10.23% [6]-shogaol, whereas the corresponding tea (Yogi Ginger Tea^®^, Eugene, OR, USA) contained only 0.142% [6]-gingerol and 0.137% [6]-shogaol [34]. As high-dose dietary supplements such as extracts are commonly consumed, it is reasonable to base calculations of C_max_ values on these formulations.

According to the ICH M12 guidance, risk assessment should be performed using the C_max,u_ values, which are corrected for plasma protein binding. C_max,u_ of [6]-gingerol was calculated as 0.231 µM (f_u_ = 0.08 [35]), and C_max,u_ of [6]-shogaol was postulated to be 0.131 µM (in the lack of literature data for f_u_, calculation was performed with the f_u_ of [6]-gingerol, given the similar structure). However, we also performed a risk assessment using the uncorrected C_max_ values, as a worst-case scenario.

### 2.7. Antiproliferative Assays

The antiproliferative properties of ginger extract, [6]-gingerol, and [6]-shogaol were determined on a panel of HEK293 cells expressing the investigated transporters using the MTT assay, as described earlier [36]. A set of human adherent cancer cell lines isolated from cervical (HeLa, SiHa, and C33A), breast (T47D, MCF7, and MDA-MB-231), oropharyngeal (UPCI-SCC-154, UPCI-SCC-131), and ovarian (A2780) cancers, and a murine fibroblast cell line (NIH/3T3) were additionally included. Briefly, all cells were cultivated in a minimal essential medium with 10% fetal bovine serum, 1% penicillin-streptomycin, and 1% non-essential amino acids at 37 °C in a humidified atmosphere containing 5% CO_2_. Cell culture media and supplements were from Lonza (Basel, Switzerland). Cells were plated into 96-well plates (5000/well, except for C33A, 10,000/well), and the next day, the tested substances or their solvents were added and incubated for 72 h. The plant extract and isolated compounds were used at concentrations of 1–90 µg/mL and 1–30 µM, respectively. After incubation, MTT solution (20 µL of 5 mg/mL) was added for 4 h, and the generated formazan crystals were dissolved in 100 μL DMSO. The absorbance was determined using a microplate reader (SPECTROstar Nano, BMG Labtech, Ortenberg, Germany), and IC_50_ values were calculated by GraphPad Prism 10.0 software. Two independent experiments were performed with five parallels for each condition.

### 2.8. Acute Toxicity Assays

Based on the results of the uptake inhibition assays and the 72 h antiproliferative assays with HEK293 cells, acute toxicity was also evaluated on the transporter-expressing cell lines that were inhibited by the constituents of *Zingiber officinale* (IC_50_ < 30 µM), using a resazurin-based cytotoxicity assay. Briefly, the cells were washed twice on a 96-well plate with 100 µL prewarmed HBSS, then incubated with 50 µL of the herbal component solution for 40 min. The test solution was then removed, and the cells were washed twice with warm HBSS. After the final aspiration, 100 µL of 70 µM resazurin solution was added to the wells, and the cells were incubated for 4 h. Fluorescence was measured on a Clariostar^plus^ microplate reader (BMG Labtech), with the appropriate wavelengths (λ_ex_= 544 nm, λ_em_ = 620 nm). Viability was calculated as A/B, where A is the background-corrected fluorescence of cells treated with the test article, and B is the background-corrected fluorescence of cells treated with vehicle control (DMSO).

## 3. Results

### 3.1. One-Point Inhibition Studies with Zingiber officinale Extract

**Figure 2:**
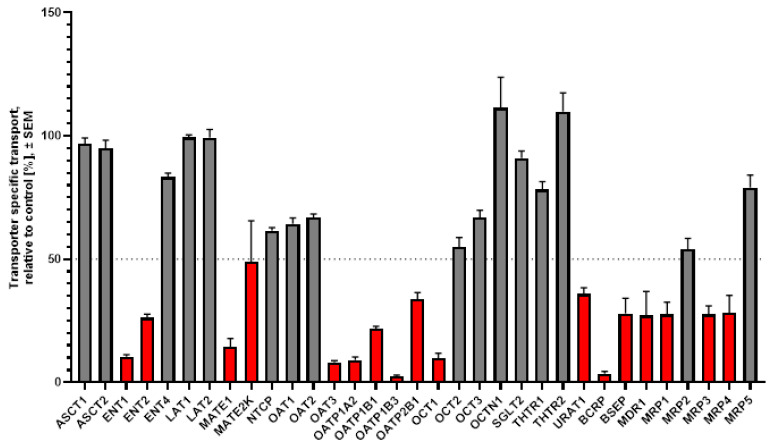
Inhibition studies with *Zingiber officinale* extract at 150 µg/mL. For each transporter shown on the horizontal axis, the bar represents the mean % inhibition ± SEM of 3 replicate measurements. Red bars below the dashed line indicate ≥50% inhibition. Grey bars above the dashed line indicate <50% inhibition.

At 150 µg/mL, ginger extract caused ≥50% inhibition of the uptake transporters ENT1, ENT2, MATE1, MATE2K, OAT3, OATP1A2, OATP1B1, OATP1B3, OATP2B1, OCT1, and URAT1, and of the efflux transporters BCRP, BSEP, MDR1, MRP1, MRP3, and MRP4 (Figure 2).

### 3.2. IC_50_ Measurements with Zingiber officinale Extract

IC_50_ measurements were conducted when the extent of inhibition exceeded 50% relative to the solvent control in the presence of 150 µg/mL *Zingiber officinale* extract. The lowest IC_50_ values were measured for OAT3 (8.395 µg/mL), BCRP (8.144 µg/mL), and ENT1 (10.76 µg/mL). Detailed results are shown in Table 2 and in Supplementary Figure S3.

### 3.3. One-Point Inhibition Studies with [6]-Gingerol and [6]-Shogaol

**Figure 3:**
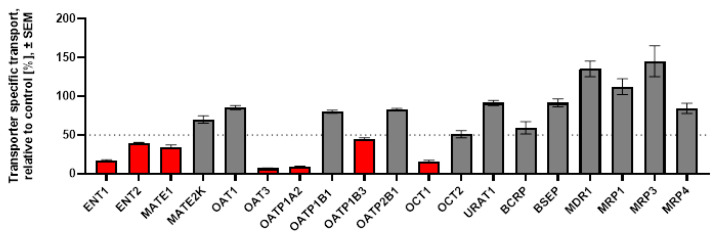
One-point inhibition studies on uptake and efflux transporters with 100 µM [6]-gingerol. For each transporter shown on the horizontal axis, the bar represents the mean % inhibition ± SEM of N = 3 measurements. Red bars below the dashed line indicate ≥50% inhibition. Grey bars above the dashed line indicate <50% inhibition.

**Figure 4:**
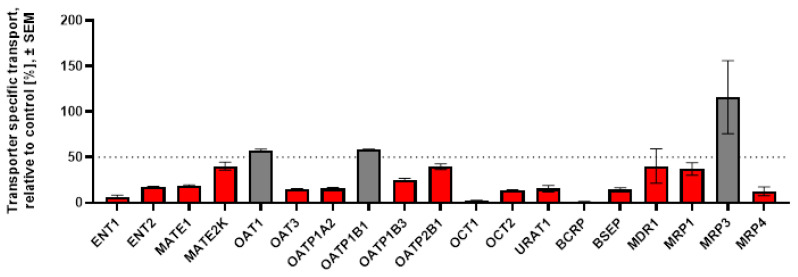
One-point inhibition studies on uptake and efflux transporters with 100 µM [6]-shogaol. For each transporter shown on the horizontal axis, the bar represents the mean % inhibition ± SEM of three replicate measurements. Red bars below the dashed line indicate ≥50% inhibition. Grey bars above the dashed line indicate <50% inhibition.

[6]-gingerol and [6]-shogaol, the most abundant active components in ginger extract, were selected for follow-up inhibitory experiments. Both were tested at 100 µM in one-point inhibition assays on all transporters that interacted with the ginger extract. OAT1 and OCT2—albeit not exhibiting interaction with the extract—were also tested with the active components, as these transporters are recommended for DDI evaluation by the ICH M12 guidance [19]. The results of these experiments are shown in Figure 3 ([6]-gingerol) and Figure 4 ([6]-shogaol).

[6]-gingerol at 100 µM inhibited 7 of the 13 uptake transporters, but none of the efflux pumps, while [6]-shogaol at 100 µM interacted with all the examined transporters except OAT1, OATP1B1, and MRP3. It is of note that both [6]-gingerol and [6]-shogaol inhibited OAT3 but not OAT1. [6]-gingerol at 100 µM was proven to be remarkably selective (15% inhibition on OAT1 vs. 93% on OAT3).

### 3.4. IC_50_ Measurements with [6]-Gingerol and [6]-Shogaol

IC_50_ measurements were conducted for all transporters that were inhibited by at least 50% at 100 μM. Detailed results are shown in Supplementary Figure S4 ([6]-gingerol), Supplementary Figure S5 ([6]-shogaol), and Table 3.

**Figure 5:**
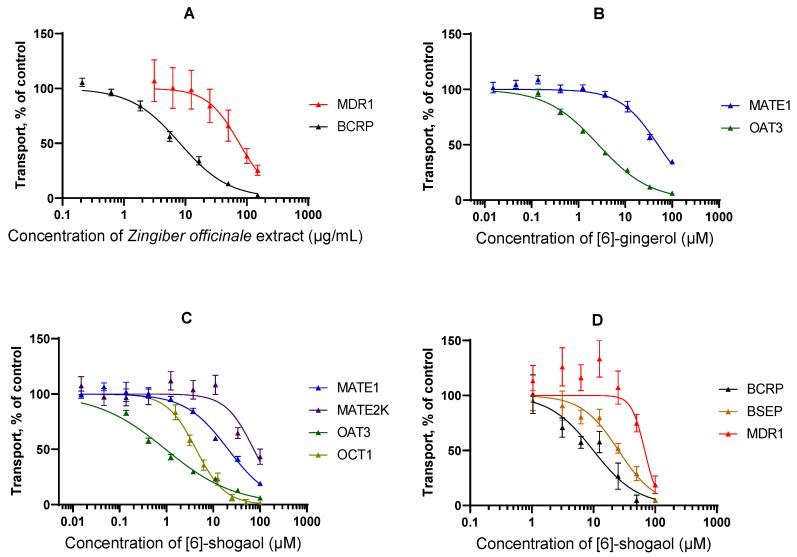
IC_50_ curves for the interactions identified as risks. (**A**) Interactions of *Zingiber officinale* extract. (**B**) Interactions of [6]-gingerol. (**C**) Interactions of [6]-shogaol with uptake transporters. (**D**) Interactions of [6]-shogaol with efflux transporters.

For interactions that were identified as potential risks in Section 3.5 (see below), IC_50_ curves are also replicated in Figure 5.

### 3.5. Risk Assessment of Transporter Interactions

For transporters expressed in renal proximal tubule cells, the measured IC_50_ values can be compared to the maximal plasma concentration C_max_, or its unbound fraction C_max,u_. A summary of our data on renal transporters is shown in Table 4 and Figure S6A. Of the listed transporters that interacted with either [6]-gingerol or [6]-shogaol, the ICH M12 guidance sets a threshold for MATE1, MATE2K, OAT3, BCRP, and MDR1: a risk of in vivo interaction is predicted if the ratio of maximal plasma concentration to the measured IC_50_ (C_max_/IC_50_) exceeds 0.1 (OAT3, OCT2), or 0.02 (MATE1, MATE2K, BCRP, MDR1).

In vivo interaction is predicted between [6]-shogaol and OAT3, even with the calculation based on the more conservative C_max,u_ value. Using the total C_max_ values as a worst-case scenario, interactions are also predicted between [6]-gingerol and MATE1 and OAT3, as well as between [6]-shogaol and MATE1, MATE2K, and the efflux transporters BCRP and MDR1. These thresholds cannot be directly applied to the remaining kidney transporters OATP1A2 and URAT1; however, it is of note that in the case of URAT1 and [6]-shogaol, IC_50_ is comparable with the maximal plasma concentration.

Regarding hepatic transporters, the ICH M12 guidance provides two different approaches. For the uptake transporter OATP1B3, the cutoff criterion is C_in,max,u_/IC_50_ ≥ 0.1, where C_in,max,u_ is the estimated unbound maximum plasma concentration of the compound at the liver inlet. For the transporters functioning as efflux pumps exporting their substrates from hepatocytes to the bile (BCRP, MDR1), the equation is the same as for some renal transporters: C_max,u_/IC_50_ ≥ 0.02 predicts in vivo interaction. Even though OATP1A2, OCT1, and BSEP are not subjects of the guidance, the uptake transporters OATP1A2 and OCT1 are evaluated using the equation provided for OATP1B1 and OATP1B3, whereas the efflux transporter BSEP was analyzed similarly to BCRP and MDR1. It is important to note that the ICH cutoff values do not officially apply to these three additional transporters. The summary of the evaluation for hepatic transporters is shown in Table 5 and Figure S6B.

In vivo interactions between [6]-shogaol and BCRP and MDR1 may be relevant, based on the C_max_/IC_50_ values. Apart from these interactions, the non-regulated BSEP also exceeds the threshold with [6]-shogaol, while OCT1’s IC_50_ is close to the C_in,max,u_ of [6]-shogaol.

For the efflux transporters expressed on the apical surface of intestinal epithelial cells (BCRP and MDR1), evaluation is meaningful not only with the active components [6]-gingerol and [6]-shogaol but also with crude ginger extract. The concentration of the extract in the small intestine is calculated as a single oral dose (2000 mg) dissolved in the typical volume of a glass of water (250 mL), according to the ICH M12 guidance. This concentration (8000 µg/mL) is compared to the measured IC_50_ values.

For the evaluation of individual active components, intestinal concentrations were calculated from the composition data of the ginger product used in the in vivo study of Yu et al.: 250 mg of Pure Encapsulations^®^ dry extract contained 6.60 mg of [6]-gingerol and 5.63 mg of [6]-shogaol [31]. At a 2000 mg dosage, this translates to 717.42 µM for [6]-gingerol and 651.81 µM for [6]-shogaol, which can be directly compared to the measured IC_50_ data.

For orally absorbed drugs, the guidance sets a threshold of 10 for the ratio of intestinal concentration C_int_ over IC_50_; thus, interaction risk is predicted if C_int_/IC_50_ exceeds 10. A summary of the evaluated data is shown in Table 6 and Figure S6C.

In vivo interaction is predicted between ginger extract and intestinal BCRP and MDR1, as well as between [6]-shogaol and BCRP.

### 3.6. Antiproliferative Properties of the Tested Substances

The 72 h cell-growth inhibitory action of ginger extract was modest against all the utilized transporter-expressing cells. Even at the highest concentration (90 μg/mL), it elicited less than or around 50% inhibition of cell proliferation (Supplementary Table S4, Figure S7). The isolated active components [6]-gingerol and [6]-shogaol were tested up to 30 μM. [6]-shogaol, but not [6]-gingerol, displayed a pronounced antiproliferative effect with typical IC_50_ values of 5–15 μM on the cervical (HeLa, SiHa, and C33A), breast (T47D, MCF7, and MDA-MB-231), oropharyngeal (UPCI-SCC-154, UPCI-SCC-131) cancer cell lines. Ovarian (A2780) and papillomavirus-negative cervical (C33A) cancer cells proved particularly sensitive, as indicated by submicromolar IC_50_ values. However, [6]-shogaol showed comparable antiproliferative activity in the noncancerous cell line NIH/3T3 cell line, suggesting the absence of a cancer-selective mechanism.

### 3.7. Acute Toxicity

As [6]-shogaol demonstrated an antiproliferative effect in our 72 h experiments, the acute toxicity of this phytochemical was measured on the cell lines expressing uptake transporters ENT1, MATE1, OATP1B3, OAT3, OCT1, OCT2, and URAT1, i.e., the ones that had an IC_50_ lower than 30 μM in our inhibitory experiments, to exclude any potential confounding effect of acute toxicity on IC_50_ results. After 40 min of treatment with 30 μM [6]-shogaol, cell viability remained above 95% in all cell lines (Figure S8), indicating that the measured inhibitory effect was not an artifact of cytotoxicity.

## 4. Discussion

Membrane transporters play pivotal roles in the uptake and efflux of endogenous and synthetic substrates. By controlling the traffic of compounds across biological barriers throughout the body, transporters are essential for the absorption, distribution, metabolism, and excretion of drugs and herbal compounds. This also implies that membrane transporters can be mediators of drug–drug and herb–drug interactions. Unlike drug–drug interactions, herb–drug interactions of even the most popular herbal products have only been studied sporadically [37].

In one of the few publications discussing the HDI liabilities of ginger, Husain et al. have evaluated the HDI potential of *Zingiber officinale* and its major constituents, focusing on interactions of ginger phytochemicals with aryl hydrocarbon receptors, the pregnane X receptor, and CYP450 enzymes, as well as the efflux transporters MDR1 and BCRP [25]. They concluded that the effects identified, i.e., strong inhibition of CYP450 enzymes, MDR1, and BCRP, carry the risk of HDIs between ginger and co-administered conventional medications. The clinical relevance of these in vitro findings is still to be confirmed. While CYP-mediated ginger HDI is supported by at least one case study [38], clinically significant transporter-mediated ginger HDIs have not yet been reported.

In our work, the effect of *Zingiber officinale* extract on a comprehensive panel of transporters, including 25 uptake and 8 efflux transporters, was tested, and the transporters that were inhibited by the extract were also evaluated with the purified single active compounds [6]-gingerol and [6]-shogaol. This allowed us to identify previously unknown ginger-transporter interactions. *Zingiber officinale* extract showed significant inhibition of 11 of the uptake and 6 of the efflux transporters, and [6]-gingerol and [6]-shogaol were also found to be potent inhibitors of 7 and 16 transporters, respectively. Although [6]-shogaol at high doses was markedly antiproliferative over a 72 h exposure period, an acute cytotoxic effect was excluded; thus, the observed inhibitory effects are confirmed to be real and transporter-specific.

Assuming high-dose (2000 mg extract/capsule) ginger supplement consumption as a worst-case scenario and applying the risk calculations laid out in the ICH M12 guidance, we conducted quantitative risk assessment for transporters with such recommendations in the document (i.e., MDR1, BCRP, OATP1B1, OATP1B3, OAT1, OAT3, OCT2, MATE1, and MATE2K). Based on this assessment, MDR1, BCRP, and OAT3 were identified as likely targets of in vivo interactions with ginger and its active constituents. Our results on MDR1 and BCRP corroborate previous reports, while OAT3 is a new finding. Additionally, we identified the hepatic transporters BSEP and OCT1 as novel suspected targets of ginger. Although BSEP and OCT1 are not explicitly mandated for investigation by the ICH M12 guidance, both are recommended for consideration on a case-by-case basis.

BCRP and MDR1 expressed in the apical membrane of intestinal epithelial cells limit the oral bioavailability of xenobiotics and drugs by actively transporting them back into the intestinal lumen, thereby reducing their absorption into systemic circulation. Their activity contributes significantly to the pharmacokinetic profiles of many therapeutic agents, and alterations in their function can lead to clinically relevant DDIs [39] and HDIs [40,41]. With C_int_/IC_50_ values well above the threshold of 10, *Zingiber officinale* extract is expected to significantly alter the intestinal absorption of BCRP/MDR1 substrate drugs co-administered with ginger.

OAT3 plays a key role in the renal elimination of various drug classes, including antibiotics, antivirals, and nonsteroidal anti-inflammatory drugs (NSAIDs), and inhibition of OAT3 by drugs and natural compounds is a well-established drug interaction mechanism [42,43]. A plethora of natural compounds, such as flavonoids, phenolic acids, and alkaloids, have been shown to potently inhibit OAT3 in vitro [42,44], and in a rat in vivo experiment, an isoflavone component of *Pueraria lobata* interfered with the OAT3-mediated renal clearance of methotrexate [45]. In our study, we measured an IC_50_ value of 0.96 μM for [6]-shogaol, which is lower than the IC_50_ of probenecid, indicating that [6]-shogaol is an extremely potent OAT3 inhibitor. For comparison, the IC_50_ of probenecid on OAT3 was approximately 20 μM in our assay system, and with a C_max,u_ of 6.77 μM (C_max_ = 14,860 μg/L [46], f_u_ = 0.13 [47]), the resulting C_max,u_/IC_50_ ratio is 0.34, which is comparable to that of [6]-shogaol (0.14). Since probenecid is the clinical control precipitant of OAT1/3-mediated DDIs [43], any OAT3 inhibitor more potent than probenecid is a likely perpetrator. Intriguingly, both [6]-gingerol and [6]-shogaol were markedly more potent inhibitors of OAT3 compared to its close cognate, OAT1, with [6]-gingerol exhibiting 6.2-fold selectivity (93% vs. 15% suppression) towards OAT3. Such inhibitor selectivity between this closely related pair of renal OATs is uncommon and remarkable, albeit not unprecedented. In a screen of 727 clinical drugs for human (h) OAT1 and hOAT3 inhibition by Duan et al., eight hOAT3-selective inhibitors were identified, and the better acceptance of bulkier substrates by the substrate binding pocket of hOAT3 was suggested as a possible explanation [48].

Albeit not mandatory, investigation of BSEP is common in drug development due to its known involvement in drug-induced liver injury (DILI). Inhibition of BSEP can lead to accumulation of toxic bile salts in hepatocytes, and DILI-causing drugs often inhibit BSEP [49], although it is evident that BSEP inhibition alone is not always predictive of DILI [50]. Morgan et al. considered drugs with BSEP IC_50_ ≤ 25 μM as potent BSEP inhibitors and pointed out that 79% (55/70) of such drugs were associated with various degrees of DILI [51]. The authors further argued that normalizing for exposure improved the prediction of DILI, and demonstrated that 95% of compounds with C_ss_/BSEP IC_50_ > 0.1, where C_ss_ (steady-state concentration) was calculated as area under the curve (AUC) over the dose interval, were associated with some extent of liver injury in humans. With its BSEP IC_50_ of 25.45 μM, [6]-shogaol lies just on the borderline, and so does its C_ss_/BSEP IC_50_ ratio with a value of 0.095 (AUC = 16.1 μg × h/mL [32], τ = 24 h). Hence, whilst ginger consumption is unlikely to be associated with clinically apparent hepatotoxicity [52], chronic exposure to high-dose ginger food supplements should be evaluated carefully.

The hepatic cation uptake transporter OCT1, too, is only recommended but not mandated for investigation. A wide range of substrates and inhibitors has been identified for this transporter, suggesting that OCT1 may represent an important mediator of clinically relevant DDIs. Nevertheless, the number of confirmed OCT1-mediated DDIs in humans remains limited [53], which can, at least in part, be explained by the fact that OCT1 shares substrate specificity with other cation transporters, such as OCT2 and MATEs. However, Matthaei et al. demonstrated that OCT1 polymorphisms affect the anti-migraine drug sumatriptan’s pharmacokinetics [54], suggesting that this drug may be a potential victim of OCT1-mediated DDIs or HDIs in vivo. Hence, OCT1 inhibition may lead to increased systemic exposure to sumatriptan and an elevated risk of adverse reactions. In our analysis, a relatively low IC_50_ of 4.29 μM and a C_in_,_max,u_/IC_50_ value of 0.089 with [6]-shogaol suggested a potential for in vivo OCT1 interactions.

Looking beyond the transporters specifically discussed in the ICH M12 guidance, our results highlight additional transporters potentially affected by ginger. Pure [6]-shogaol inhibited URAT1, the principal urate reabsorption transporter of renal proximal tubule cells, with a potency on a par with the selective urate reabsorption inhibitor lesinurad (IC_50_: 5.89 μM and 3.53 μM, respectively [55]). While this might not be a safety concern *per se*, potential synergy should be considered when ginger is co-administered with urate-lowering therapy. The ubiquitously expressed equilibrative nucleoside transporter 1 (ENT1), too, was inhibited by [6]-shogaol with a relatively low IC_50_ of 11.6 μM. ENT1 mediates the cellular import of many nucleoside/nucleotide analogs applied in antiviral and anticancer therapy, and ENT1 inhibitors have been documented to interfere with the targeting and disposition of these clinically important medications [56]. Considering its C_max,u_/IC_50_ of 0.011 (C_max,total_/IC_50_ = 0.14), [6]-shogaol is expected to pose a moderate but non-negligible risk of ENT1 HDI, with an interaction potential similar to that of known ENT1 blockers, such as tyrosine kinase inhibitors [57].

In summary, based on in vitro determined IC_50_ values and standard risk calculations, we suggest that prolonged consumption of ginger supplements, especially at high doses, may precipitate transporter-mediated herb–drug interactions. Beyond the previously acknowledged inhibitory effects of ginger components on MDR1 and BCRP, we have identified additional transporters as potential targets of ginger HDI. Specifically, active components of ginger may interfere with renal drug disposition by inhibiting OAT3, as well as with hepatic drug disposition by inhibiting OCT1; further, they may potentiate urate-lowering therapies by inhibiting URAT1 and modulate the pharmacokinetics of nucleoside/nucleotide drugs by inhibiting ENT1. Inhibition of OAT3 and OCT1 by ginger may be of particular interest for patients taking pharmaceuticals with narrow therapeutic windows, because for these drugs, even a minor obstruction of elimination and consequent elevation of systemic exposure may be sufficient to precipitate adverse effects. Evidently, whether these in vitro identified interactions are significant in vivo depends on a myriad of unknown pharmacokinetic factors like the tissue distribution, local disposition, or individual variation in the bioavailability of ginger components. Also, while [6]-shogaol displayed a potent and rather indiscriminate antiproliferative effect in vitro, it remains to be clarified whether and how this correlates with ginger’s purported in vivo antitumor effect. Since ginger as a food supplement is widely consumed but poorly regulated, it is essential to raise awareness of its potential for drug interactions and to follow up on suspected effects in in vivo studies.

## Figures and Tables

**Table 1:** Literature data on [6]-gingerol and [6]-shogaol plasma concentrations in healthy humans. C_max_ is recorded as µg/mL. C_max_ (uncon)—highest plasma concentration of the unconjugated forms C_max_ (total)—highest plasma concentration measured after incubation with β-glucuronidase and sulfatase, and thus represents combined conjugates. Highest reported C_max_ values for each compound are highlighted in bold. ND—not detectable.

	Zick et al.		Yu et al.		Levita et al.	
	C_max_ (Uncon)	C_max_ (Total)	C_max_ (Uncon)	C_max_ (Total)	C_max_ (Uncon)	C_max_ (Total)
[6]-gingerol	ND	**0.85**	ND	0.71	-	-
[6]-shogaol	ND	0.15	0.011	0.14	**0.453**	-

**Table 2:** Best-fit IC_50_ values and profile likelihood of inhibition with *Zingiber officinale* extract. IC_50_ values are color-coded from red (lowest) to green (highest).

		Best-Fit Values (µg/mL)	95% Confidence Interval
Uptake transporters	ENT1	10.76	8.999 to 12.87
	ENT2	48.76	44.92 to 53.03
	OAT3	8.395	7.285 to 9.656
	OATP1A2	18.95	17.84 to 20.13
	OATP1B1	32.75	26.88 to 40.22
	OATP1B3	24.29	22.02 to 26.78
	OATP2B1	67.06	45.76 to 108.7
	MATE1	24.96	18.52 to 33.96
	MATE2K	147.7	117.0 to 227.3
	OCT1	18.61	16.60 to 20.87
	URAT1	55.53	45.13 to 69.99
Efflux transporters	BCRP	8.144	6.477 to 10.26
	BSEP	43.9	31.52 to 63.46
	MDR1	75.75	64.61 to 88.98
	MRP1	60.87	40.33 to 95.67
	MRP3	67.06	48.61 to 99.26
	MRP4	72.54	59.55 to 88.47

**Table 3:** Best-fit IC_50_ values and profile likelihood of inhibition with [6]-gingerol and [6]-shogaol. IC_50_ values are color-coded from red (lowest) to green (highest).

			Best-Fit Values (µM)	95% Confidence Interval
[6]-gingerol	Uptake transporters	ENT1	25.89	20.68 to 32.54
		ENT2	75.55	71.40 to 80.24
		MATE1	49.74	38.63 to 66.01
		OAT3	2.655	2.153 to 3.276
		OATP1A2	41.33	37.03 to 46.22
		OATP1B3	76.66	63.13 to 98.53
		OCT1	13.75	12.73 to 14.84
[6]-shogaol	Uptake transporters	ENT1	11.62	9.711 to 13.91
		ENT2	38.2	27.40 to 55.49
		MATE1	20.75	16.83 to 25.72
		MATE2K	72.99	46.21 to 140.0
		OAT3	0.956	0.651 to 1.378
		OATP1A2	35	27.71 to 44.76
		OATP1B3	19.2	17.07 to 21.63
		OATP2B1	90.99	66.09 to 157.3
		OCT1	4.287	3.796 to 4.832
		OCT2	24.43	17.93 to 33.55
		URAT1	5.887	3.539 to 9.732
	Efflux transporters	BCRP	9.931	6.072 to 15.68
		BSEP	25.45	19.93 to 32.28
		MDR1	67.64	41.55 to 141.0
		MRP1	73.19	60.69 to 91.81
		MRP4	23.81	16.69 to 33.89

**Table 4:** In vivo risk assessment for the renal transporters that interacted with [6]-gingerol or [6]-shogaol. Transporters marked with * are not expected to be evaluated as per the ICH M12 guidance. Red case: possible in vivo interactions according to the ICH M12 guidance, boldface: possible in vivo interaction predicted based on the ICH M12 cutoff, using the total C_max_ values. [6]-gingerol: C_max_ = 2.887 µM, C_max,u_ = 0.231 µM [6]-shogaol: C_max_ = 1.640 µM, C_max,u_ = 0.131 µM.

	[6]-Gingerol			[6]-Shogaol		
Transporter	IC_50_ (µM)	C_max_/IC_50_	C_max,u_/IC_50_	IC_50_ (µM)	C_max_/IC_50_	C_max,u_/IC_50_
MATE1	49.74	**0.058**	0.005	20.75	**0.079**	0.006
MATE2K	-	-	-	72.99	**0.022**	0.002
OAT3	2.66	**1.09**	0.087	0.96	**1.717**	**0.137**
OCT2	-	-	-	24.43	0.067	0.005
BCRP	-	-	-	9.93	**0.165**	0.013
MDR1	-	-	-	67.64	**0.024**	0.002
OATP1A2 *	41.33	0.07	0.006	35.00	0.047	0.004
URAT1 *	-	-	-	5.89	0.279	0.022

**Table 5:** In vivo risk assessment for the hepatic transporters that interact with [6]-gingerol or [6]-shogaol. Transporters marked with * are not specifically addressed by the ICH M12 guidance. Boldface: possible in vivo interaction predicted based on the ICH M12 cutoff, using the total C_max_ values. [6]-gingerol: C_max_ = 2.887 µM, C_max,u_ = 0.231 µM, C_in,max,u_ = 1.221 µM. [6]-shogaol: C_max_ = 1.640 µM, C_max,u_ = 0.131 µM, C_in,max,u_ = 1.030 µM. N/A, not applicable.

	[6]-Gingerol				[6]-Shogaol			
Transporter	IC_50_ (µM)	C_max_/IC_50_	C_max,u_/IC_50_	C_in,max,u_/IC_50_	IC_50_ (µM)	C_max_/IC_50_	C_max,u_/IC_50_	C_in,max,u_/IC_50_
MDR1	-	-	-	-	67.64	**0.024**	0.002	N/A
BCRP	-	-	-	-	9.931	**0.165**	0.013	N/A
BSEP *	-	-	-	-	25.45	0.064	0.005	N/A
OATP1B3	-	-	-	-	19.20	N/A	N/A	0.054
OATP1A2 *	41.33	N/A	N/A	0.030	35.00	N/A	N/A	0.029
OCT1 *	13.75	N/A	N/A	0.089	4.287	N/A	N/A	0.240

**Table 6:** In vivo risk assessment for the intestinal transporters that interact with ginger extract. C_int_ = 8000 µg/mL for the extract, 717.42 µM for [6]-gingerol, and 651.81 µM for [6]-shogaol. C_int_/IC_50_ ≥ 10 (marked with red) predicts possible in vivo interactions.

	Extract		[6]-Gingerol		[6]-Shogaol	
Transporter	IC_50_ (µg/mL)	C_int_/IC_50_	IC_50_ (µM)	C_int_/IC_50_	IC_50_ (µM)	C_int_/IC_50_
BCRP	8.144	982.3	-	-	9.93	65.6
MDR1	75.75	105.6	-	-	67.64	9.64

## Data Availability

The data presented in this study are available on request from the corresponding author. The data are not publicly available due to the company’s participation.

## Supplementary Materials

The following supporting information can be downloaded at: https://www.mdpi.com/article/10.3390/pharmaceutics18020149/s1, Figure S1: HPLC chromatograms and UV spectra of [6]-gingerol + [6]-shogaol and 95% MeOH extract of ginger rhizome; Figure S2: Calibration curves of [6]-gingerol and [6]-shogaol; Figure S3: IC_50_ studies on different uptake and efflux transporters with *Zingiber officinale* extract; Figure S4: IC_50_ studies with [6]-gingerol; Figure S5: IC_50_ studies on uptake and efflux transporters with [6]-shogaol; Figure S6: Summary of risk assessment of the transporter interactions; Figure S7: Antiproliferative effect of [6]-shogaol on cancer cell lines; Figure S8: The acute effect of a 40-min treatment with 30 μM [6]-shogaol on the viability of cell lines whose proliferation was negatively affected by a 72-h treatment; Table S1: List of the transporters examined in the study; Table S2: Assay parameters for uptake transporter assays; Table S3: Assay parameters for vesicular transport assays; Table S4: Antiproliferative properties of ginger extract and the isolated natural products. References cited in the supplement: [58,59,60,61,62,63,64,65,66,67,68,69,70,71,72,73,74,75,76,77].

## Abbreviations

The following abbreviations are used in this manuscript:

SLC	Solute carrier
ABC	ATP-binding cassette
HDI	Herb–drug interaction
MDR1	Multidrug resistance 1
BCRP	Breast cancer resistance protein
OAT	Organic anion transporter
URAT	Urate transporter
OCT	Organic cation transporter
BSEP	Bile salt export pump
ENT	Equilibrative nucleoside transporter
DDI	Drug–drug interaction
MRP	Multidrug resistance protein
OATP	Organic anion transporting polypeptide
MATE	Multidrug and toxin extrusion transporter
CYP	Cytochrome P450
HPLC	High-Pressure Liquid Chromatography
LOD	Limit of detection
LOQ	Limit of quantification
PTFE	Polytetrafluoroethylene
DAD	Diode array detector
SD	Standard deviation
HEK293	Human embryonic kidney 293
MDCKII	Madin-Darby canine kidney strain II
DMEM	Dulbecco’s Modified Eagle Medium
FBS	Fetal bovine serum
E3S	Estrone-3-sulfate
NMQ	N-methyl-quinidine
CCK-8	Cholecystokinin octapeptide
MPP^+^	1-methyl-4-phenylpyridinium
DHEAS	Dehydroepiandrosterone sulfate
E_2_17βG	Estradiol 17β-D-glucuronide
TC	Taurocholic acid
AMG	α-D-glucopyranoside
TEA	Tetraethylammonium
HBSS	Hanks’ Balanced Salt Solution
KH	Krebs–Henseleit
ICH	International Council for Harmonization
DMSO	Dimethyl sulfoxide
DILI	Drug-induced liver injury
AUC	Area under the curve
