# Supplementary material for: Ginger and Its Purified Major Components Inhibit Clinically Relevant Uptake and Efflux Transporters In Vitro
Source: Pharmaceutics. 2026 Jan 23;18(2):149. doi: 10.3390/pharmaceutics18020149 (PMC12944454; doi:10.3390/pharmaceutics18020149)
Supplement: Supplementary file 1 [file pharmaceutics-18-00149-s001.zip › pharmaceutics-4043199-supplementary.pdf]

Table S1. List of the transporters examined in the study.

Transporter	Localization	Key substrates **(drugs)**	References
ASCT1	Brain, kidney	Serine, alanine	[1]
ASCT2	Intestine, kidney, brain	Glutamine	[1]
ENT1	All tissues	Adenosine, **gemcitabine**	[2]
ENT2	All tissues	Adenosine, **fludarabine**	[2]
ENT4	Brain, heart	Adenosine, **metformin**	[2], [3]
LAT1	Brain, placenta, tumors	Leucine, isoleucine, **levodopa**	[4], [5]
LAT2	Kidney, brain	Leucine, isoleucine	[4]
MATE1	Liver, kidney	**Metformin**	[6]
MATE2K	Kidney	**Metformin**	[6]
NTCP	Liver	Taurocholate, **rosuvastatin**	[7]
OAT1	Kidney	Prostaglandin E2, **methotrexate**	[8], [9]
OAT2	Liver, kidney	Estrone-3-sulfate, **diclofenac**	[8]
OAT3	Kidney	**Pravastatin, methotrexate**	[8]
OATP1A2	Liver, brain	Bile salts, **methotrexate**	[8]
OATP1B1	Liver	Bilirubin, **statins**	[8]
OATP1B3	Liver	Bilirubin, **statins**	[8]
OATP2B1	Liver, intestine	Estrone-3-sulfate, **statins**	[8]
OCT1	Liver	Thiamine, **metformin**	[10]
OCT2	Kidney	Tetraethylammonium, **metformin**	[10]
OCT3	Placenta, liver	Dopamine, **metformin**	[10]
OCTN1	Kidney, brain	Ergothioneine	[10]
SGLT2	Kidney	Glucose	[11]
THTR1	Intestine, brain	Thiamine	[12]
THTR2	Brain, placenta	Thiamine, **metformin**	[12], [13]
URAT1	Kidney	Uric acid	[14]
BCRP	Intestine, kidney, liver, brain	**Topotecan, mitoxantrone**	[15]
BSEP	Liver	Bile acids, **rifampin**	[16]
MDR1 (P-gp)	Intestine, kidney, liver, brain	**Digoxin, paclitaxel**	[17]
MRP1	All tissues	Glutathione conjugates, **vincristine**	[18]
MRP2	Liver, kidney	Bilirubin, **methotrexate**	[18]
MRP3	Liver, kidney, intestine	Glucuronide conjugates, **etoposide**	[18]
MRP4	Kidney, brain	Nucleotide analogs, **tenofovir**	[18], [19]
MRP5	All tissues	Nucleotide analogs, **PMEA (9-(2-phosphonomethoxyethyl)adenine)**	[18], [20]

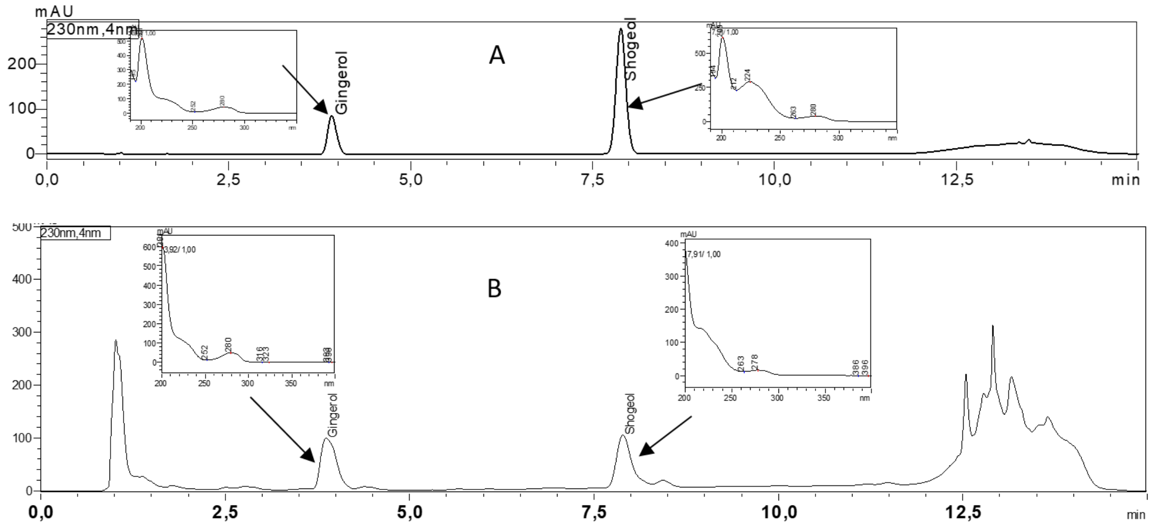

Figure S1. HPLC chromatograms and UV spectra of [6]-gingerol + [6]-shogaol (A), and 95% MeOH extract of ginger rhizome (B) [column: Kinetex C-8 column (100 Å, 150×4.60 mm, 5 μm), eluent: gradient system of MeOH and H_2O with 0.1% phosphoric acid; flow rate: 1.5 mL/min; detection at 230 nm].

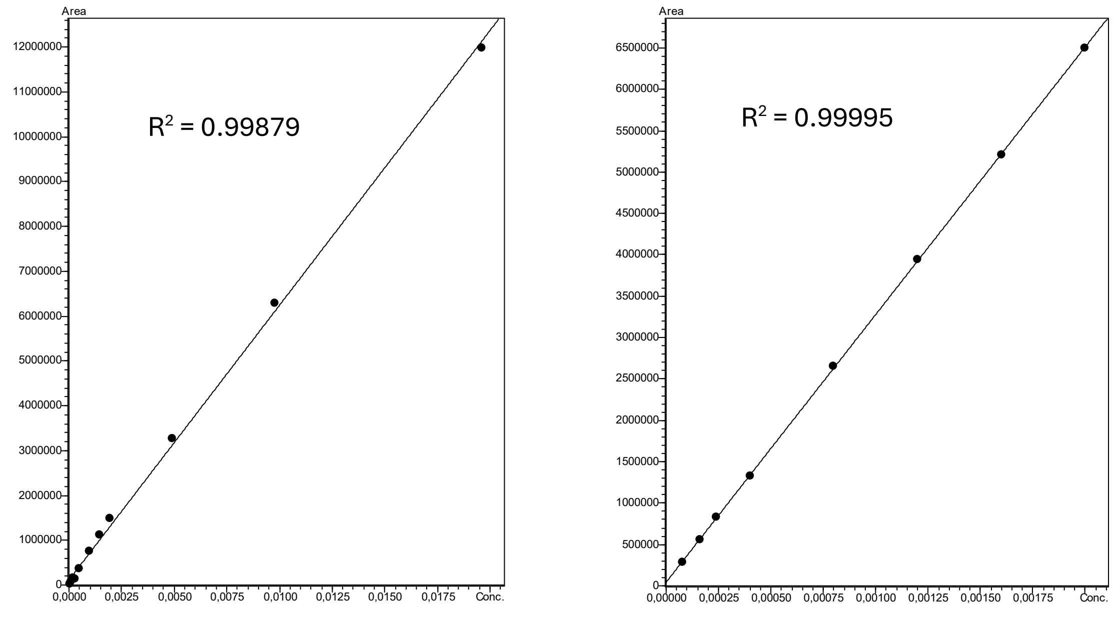

A B

Figure S2. Calibration curves of [6]-gingerol (A) and [6]-shogaol (B).

Table S2. Assay parameters for uptake transporter assays. Abbreviations: MPP^+: 1-methyl-4-phenylpyridinium, TC: taurocholate, E3S: estrone-3-sulfate, CCK-8: cholecystokinin fragment 26-33 amide, TEA: tetraethylammonium. Buffers: **HBSS**, Hanks' Balanced Salt Solution, 10×, Gibco 14065056 (ThermoFisher, Waltham, MA, USA), diluted to 1× with deionized water, pH set to 7,4; **HBSS w/o Cl^-**, chloride-free HBSS: 125 mM sodium gluconate, 4.8 mM potassium gluconate, 1.2 mM dibasic potassium phosphate, 1.3 mM calcium gluconate, 1.2 mM magnesium sulfate, 5.6 mM D-glucose, and 25 mM HEPES, pH set to 7.4; **HBSS w/o glucose**, glucose-free HBSS: 4.17 mM sodium bicarbonate, 5.37 mM potassium chloride, 0.44 mM monobasic potassium phosphate, 0.81 mM magnesium sulfate, 1.5 mM calcium chloride, 137 mM sodium chloride, and 0.33 mM dibasic sodium phosphate, pH set to 7.4; **KH**, Krebs-Henseleit buffer: 24 mM sodium bicarbonate, 4.83 mM potassium chloride, 0.96 mM dibasic potassium phosphate, 110 mM sodium chloride, 1.22 mM magnesium sulfate, 1.53 mM calcium chloride, 5 mM D-glucose, and 13 mM HEPES, pH set to 7.4; **KH w/o Na^+**, sodium-free Krebs-Henseleit buffer: 0.424% v/v choline bicarbonate, 4.83 mM potassium chloride, 0.96 mM dibasic potassium phosphate, 110 mM choline chloride, 1.22 mM magnesium sulfate, 1.53 mM calcium chloride, 5 mM D-glucose, and 13 mM HEPES, pH set to 7.4.

NCBI Reference Sequence	Transporter	Assay duration (min)	Substrate	Substrate concentration (μM)	Incubation temperature (°C)	Buffer	pH
NM_003038.5	ASCT1	1	Serine	1	25	KH	7.4
NM_005628.3	ASCT2	1	Serine	1	25	KH	7.4
NM_001078177	ENT1	1	Uridine	1	37	KH	7.4
NM_001532.2	ENT2	2	Adenosine	1	37	KH	7.4
NM_001040661.1	ENT4	2	MPP^+	10	37	HBSS	6.6
NM_003486.6	LAT1	1	Leucine	1	37	KH w/o Na^+	7.4
NM_012244.4	LAT2	1	Leucine	1	37	KH w/o Na^+	7.4
NM_018242.3	MATE1	15	Metformin	10	37	KH	8
NM_001099646.2	MATE2K	5	Metformin	10	37	KH	8
NM_003049.3	NTCP	2	TC	2	37	HBSS	7.4
BC033682.1	OAT1	2	Tenofovir	5	37	HBSS	7.4
NM_006672.3	OAT2	15	Uric acid	25	37	HBSS	7.4
BC022387	OAT3	3	E3S	1	37	HBSS	7.4
NM_134431	OATP1A2	1	E3S	1	37	HBSS	7.4
NM_006446.4	OATP1B1	2	E3S	0.1	37	HBSS	7.4
NM_019844	OATP1B3	2	CCK-8	1	37	HBSS	7.4
NM_007256	OATP2B1	2	E3S	1	37	HBSS	7.4
BC126364.1	OCT1	5	Metformin	10	37	HBSS	7.4
NM_003058	OCT2	1	Metformin	10	37	HBSS	7.4
NM_021977	OCT3	3	MPP^+	0.02	37	KH	7.4
NM_003059.3	OCTN1	8	TEA	10	37	HBSS	7.4

AJ133127.1	SGLT2	10	AMG	1	37	HBSS w/o glucose	7.4
NM_006996	THTR1	3	Thiamine	0.025	37	HBSS	7.4
NM_025243	THTR2	3	Thiamine	0.025	37	HBSS	7.4
BC053348	URAT1	10	Uric acid	20	37	HBSS w/o Cl^-	7.4

Table S3. Assay parameters for vesicular transport assays. Abbreviations: E3S: estrone-3-sulfate, TC: taurocholate, NMQ: N-methylquinidine, E217βG: β-estradiol-17-(β-D-glucuronide), DHEAS: dehydroepiandrosterone sulfate, CDCF: 5(6)-carboxy-2′,7-dichlorofluorescein B. **Buffers: ‘A’**: transport buffer without sucrose: 46.5 mM MOPS-TRIS, 65.1 mM KCl and 7 mM $MgCl_2$ in purified water; **‘B’**: transport buffer for BSEP: 2 mM HEPES-Tris, 50 mM sucrose, 100 mM KNO_3, 10 mM $Mg(NO_3)_2$ in purified water; **‘C’**: transport buffer with sucrose: 10 mM Tris-HCl, 250 mM sucrose, 10 mM $MgCl_2$ in purified water.

NCBI Reference Sequence	Transporter	Assay duration (min)	Substrate	Substrate concentration (μM)	Incubation temperature (°C)	Buffer
NM_004827.2	BCRP	1	E3S	1	32	‘A’
NM_003742.2	BSEP	5	TC	0.2	37	‘B’
NM_000927.4	MDR1	1	NMQ	1	32	‘A’
NM_004996	MRP1	5	E217βG	0.5	37	‘A’
NM_000392.4	MRP2	5	E217βG	100	37	‘C’
BC137347.1	MRP3	10	E217βG	10	37	‘C’
NM_005845.4	MRP4	1.5	DHEAS	0.5	32	‘C’
NM_005688.2	MRP5	5	CDCF	10	37	‘A’

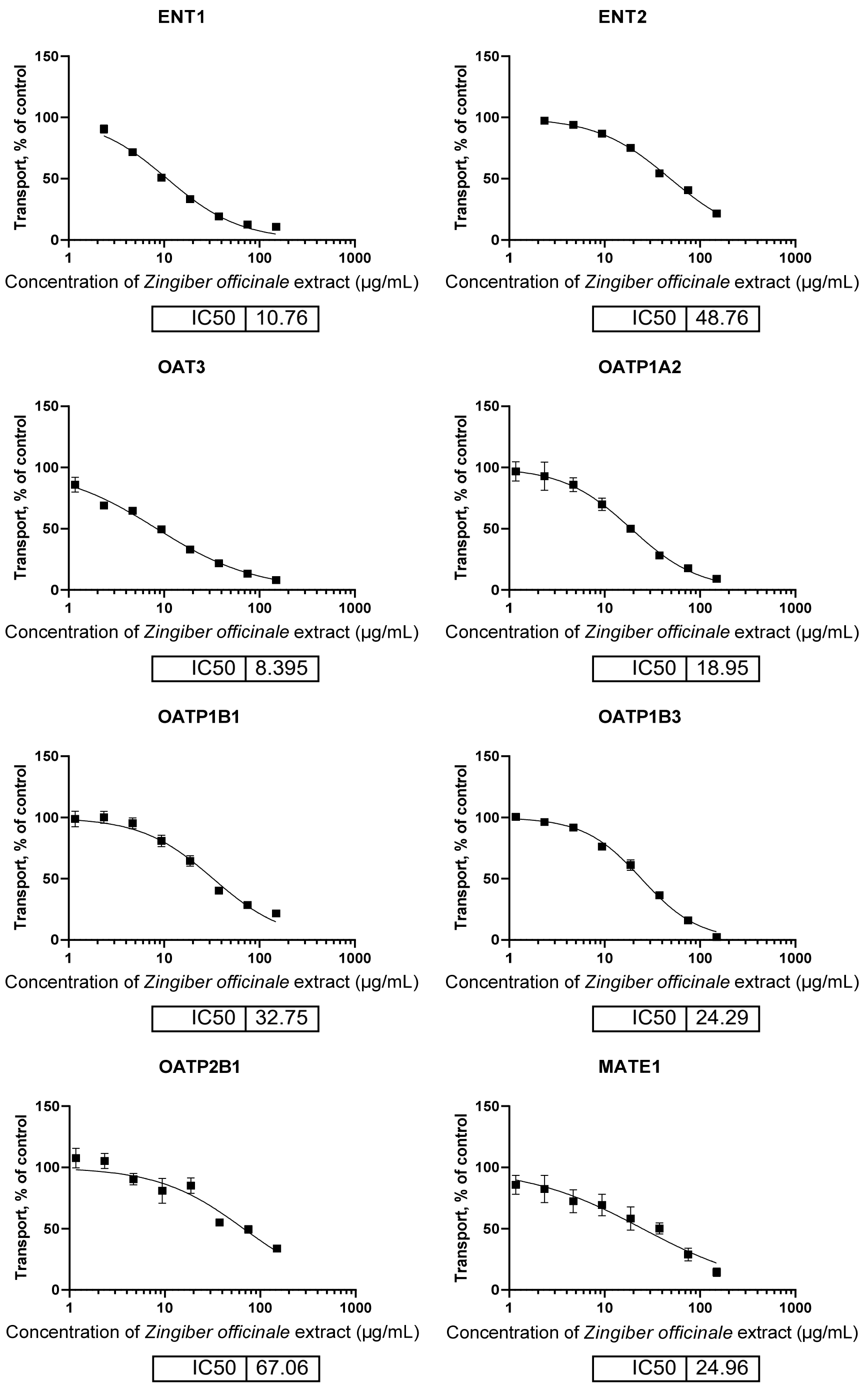
ENT1
Transport, % of control
150
100
50
0
1
10
100
1000
Concentration of *Zingiber officinale* extract (μg/mL)
IC50 10.76
ENT2
Transport, % of control
Concentration of *Zingiber officinale* extract (μg/mL)
IC50 48.76
OAT3
Transport, % of control
Concentration of *Zingiber officinale* extract (μg/mL)
IC50 8.395
OATP1A2
Transport, % of control
Concentration of *Zingiber officinale* extract (μg/mL)
IC50 18.95
OATP1B1
Transport, % of control
Concentration of *Zingiber officinale* extract (μg/mL)
IC50 32.75
OATP1B3
Transport, % of control
Concentration of *Zingiber officinale* extract (μg/mL)
IC50 24.29
OATP2B1
Transport, % of control
Concentration of *Zingiber officinale* extract (μg/mL)
IC50 67.06
MATE1
Transport, % of control
Concentration of *Zingiber officinale* extract (μg/mL)
IC50 24.96

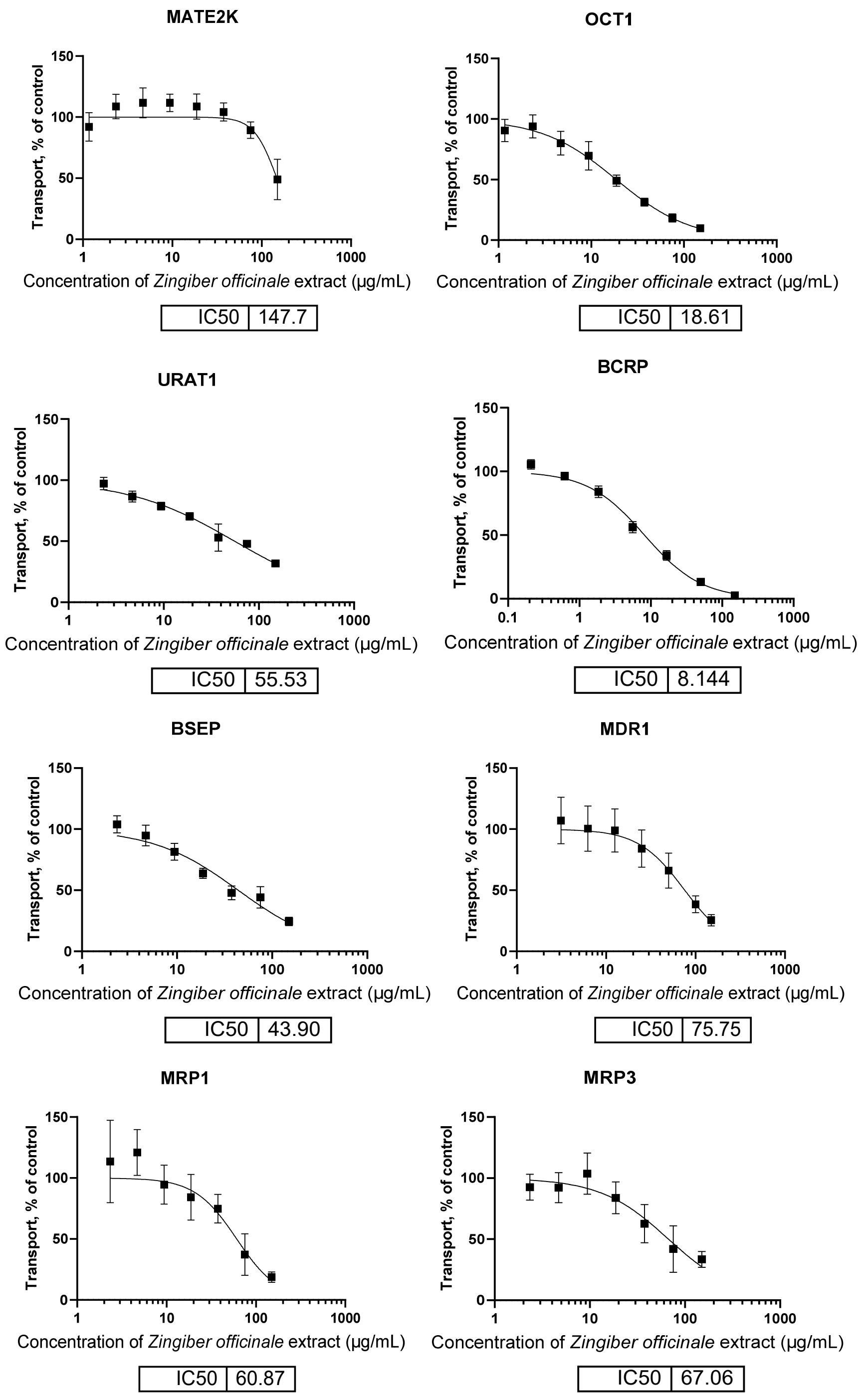
MATE2K
Transport, % of control
Concentration of Zingiber officinale extract (µg/mL)
IC50 147.7
OCT1
Transport, % of control
Concentration of Zingiber officinale extract (µg/mL)
IC50 18.61
URAT1
Transport, % of control
Concentration of Zingiber officinale extract (µg/mL)
IC50 55.53
BCRP
Transport, % of control
Concentration of Zingiber officinale extract (µg/mL)
IC50 8.144
BSEP
Transport, % of control
Concentration of Zingiber officinale extract (µg/mL)
IC50 43.90
MDR1
Transport, % of control
Concentration of Zingiber officinale extract (µg/mL)
IC50 75.75
MRP1
Transport, % of control
Concentration of Zingiber officinale extract (µg/mL)
IC50 60.87
MRP3
Transport, % of control
Concentration of Zingiber officinale extract (µg/mL)
IC50 67.06

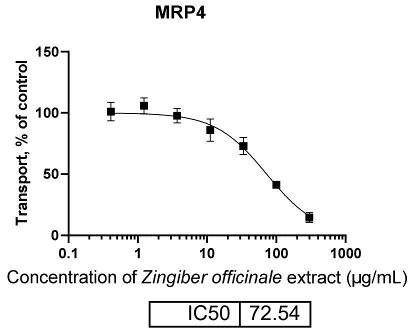

Figure S3. IC_{50} studies on different uptake and efflux transporters with *Zingiber officinale* extract

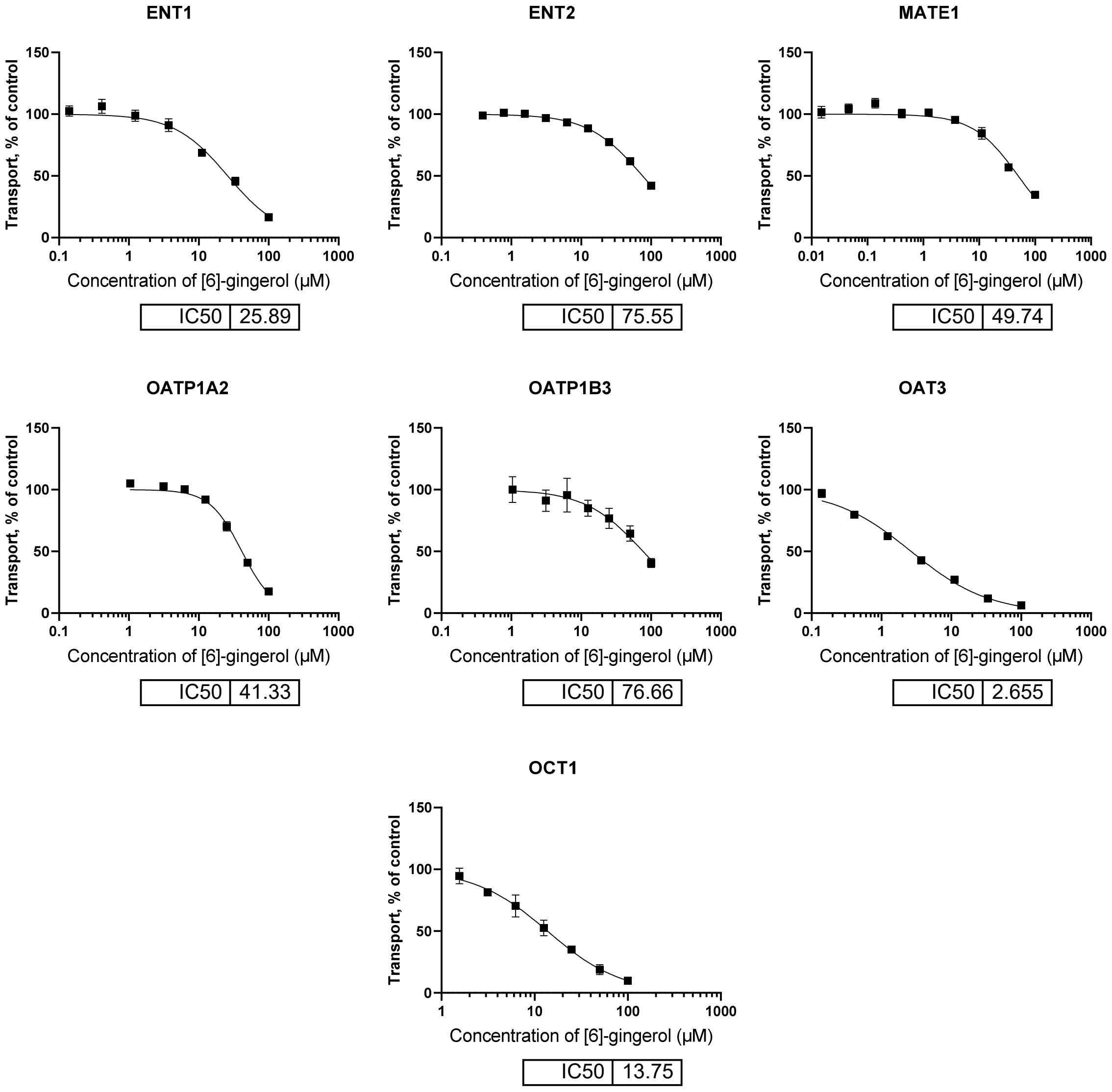

Figure S4. IC_{50} studies with [6]-gingerol.

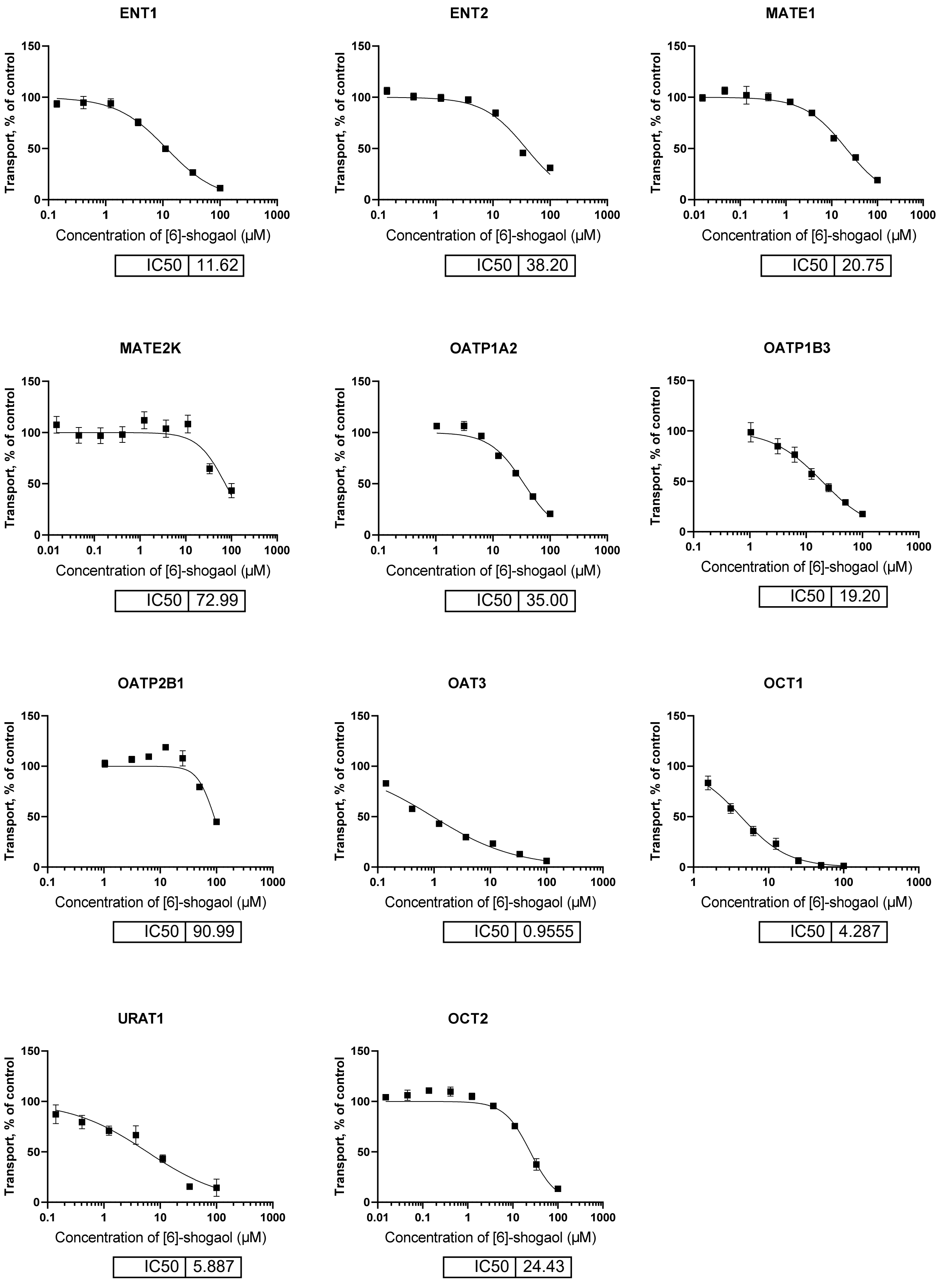
ENT1
ENT2
MATE1
MATE2K
OATP1A2
OATP1B3
OATP2B1
OAT3
OCT1
URAT1
OCT2
Transport, % of control
Concentration of [6]-shogaol (μM)
IC50 11.62
IC50 38.20
IC50 20.75
IC50 72.99
IC50 35.00
IC50 19.20
IC50 90.99
IC50 0.9555
IC50 4.287
IC50 5.887
IC50 24.43

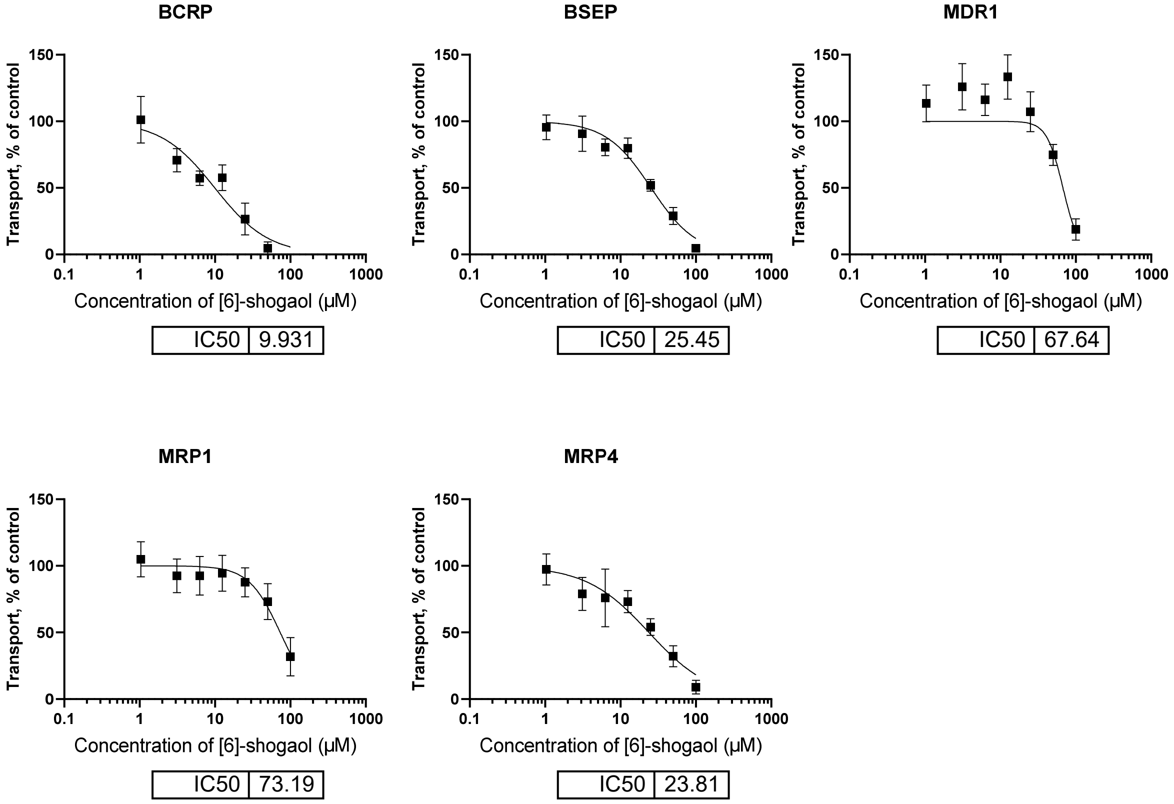

Figure S5. IC_{50} studies on uptake and efflux transporters with [6]-shogaol.

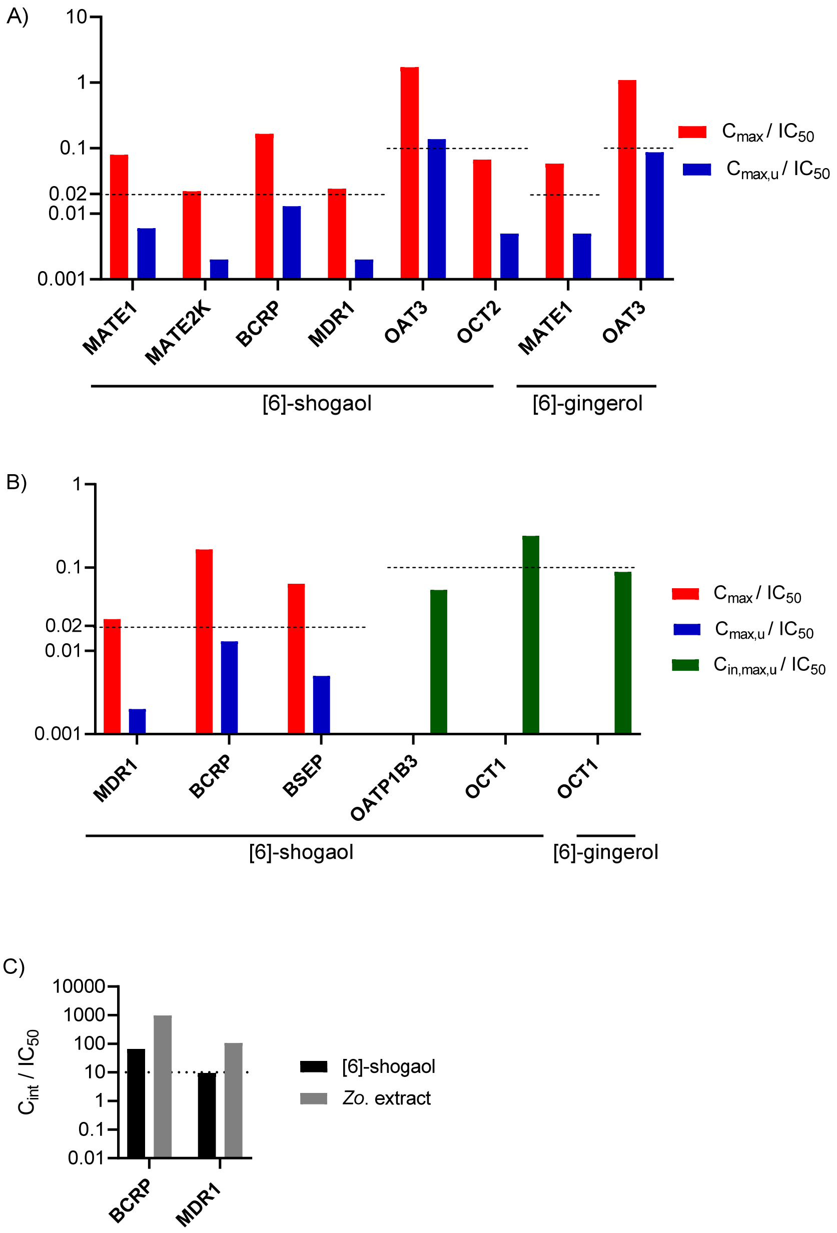

Figure S6. Summary of risk assessment of the transporter interactions. **A)** Renal transporters. The risk assessment was based on the maximum plasma concentrations, with (blue) and without (red) f_u-correction. A cutoff of 0.02 was applied for MATE1, MATE2K, BCRP and MDR1; and a cutoff of 0.1 was applied for OAT3 and OCT2 (dashed lines). **B)** Hepatic transporters. For the efflux transporters MDR1, BCRP and BSEP, risk assessment was performed based on the systemic plasma concentrations and a cutoff of 0.02 was applied, whereas for the uptake transporters OATP1B3 and OCT1, the liver inlet concentrations were used, and the cutoff was 0.1 (green). **C)** Intestinal transporters. A cutoff of 10 was applied and the calculation was performed using the corresponding intestinal concentrations.

Table S4. Antiproliferative properties of ginger extract and the isolated natural products.

Cell line	Extract concentration	Inhibition of cell growth (%) ± SEM	Natural product concentration	Inhibition of cell growth (%) ± SEM [calculated IC_{50} (μM)]	
				[6]-gingerol	[6]-shogaol
HEK293-OAT1	60 μg/mL	37.48 ± 1.44	10 μM	–[1]	87.68 ± 0.30
	90 μg/mL	46.85 ± 2.59	30 μM	–	97.28 ± 0.10 [5.46]
HEK293-OCT1	60 μg/mL	34.41 ± 1.77	10 μM	12.01 ± 1.99	75.82 ± 1.35
	90 μg/mL	48.86 ± 2.95	30 μM	14.46 ± 3.84	95.01 ± 1.22 [6.61]
HEK293-MDR1	60 μg/mL	41.84 ± 1.70	10 μM	–	65.34 ± 0.77
	90 μg/mL	48.04 ± 1.76	30 μM	–	96.08 ± 0.48 [8.69]
HEK293-Mock LV	60 μg/mL	16.37 ± 1.03	10 μM	15.89 ± 1.67	–
	90 μg/mL	20.69 ± 1.06	30 μM	19.59 ± 3.05	92.28 ± 1.72 [17.66]
HEK293-Mock B LV	60 μg/mL	26.42 ± 3.36	10 μM	–	–
	90 μg/mL	31.85 ± 1.16	30 μM	15.44 ± 2.35	55.40 ± 3.58 [28.62]
HEK293-OATPB3	60 μg/mL	43.35 ± 2.15	10 μM	–	47.52 ± 1.23
	90 μg/mL	55.38 ± 2.61	30 μM	–	93.05 ± 2.21 [10.47]
HEK293-OATP1A2	60 μg/mL	28.20 ± 1.90	10 μM	–	68.92 ± 1.40
	90 μg/mL	39.37 ± 1.66	30 μM	–	95.04 ± 0.35 [7.74]
HEK293-OATP1B1	60 μg/mL	41.37 ± 2.92	10 μM	26.65 ± 1.78	61.84 ± 1.70
	90 μg/mL	52.39 ± 0.91	30 μM	30.33 ± 3.72	86.60 ± 1.09 [8.67]
HEK293-OATP2B1	60 μg/mL	42.73 ± 1.01	10 μM	–	60.34 ± 2.08
	90 μg/mL	57.23 ± 2.12	30 μM	–	88.25 ± 0.52 [8.44]
HEK293-OCT2	60 μg/mL	–	10 μM	22.85 ± 2.44	58.59 ± 1.11
	90 μg/mL	38.53 ± 1.51	30 μM	23.81 ± 2.46	90.09 ± 0.08 [8.54]
HEK293-OAT3	60 μg/mL	33.86 ± 1.66	10 μM	–	63.79 ± 0.64

	90 µg/mL	46.49 ± 1.09	30 µM	10.31 ± 2.28	99.84 ± 0.15<br>[7.23]
HEK293-BRCP	60 µg/mL	36.10 ± 2.74	10 µM	–	47.44 ± 1.31
	90 µg/mL	41.01 ± 2.07	30 µM	20.94 ± 1.37	94.78 ± 0.17<br>[10.38]
UPCI-SCC-131	60 µg/mL	46.40 ± 2.30	10 µM	–	44.25 ± 0.50
	90 µg/mL	62.64 ± 2.99	30 µM	22.69 ± 2.53	95.42 ± 0.27<br>[10.65]
UPCI-SCC-154	60 µg/mL	41.89 ± 1.15	10 µM	17.07 ± 1.21	54.66 ± 3.01
	90 µg/mL	46.20 ± 1.09	30 µM	22.30 ± 1.50	84.20 ± 1.75<br>[8.25]
MDA-MB-231	60 µg/mL	23.74 ± 0.95	10 µM	–	23.27 ± 1.08
	90 µg/mL	25.65 ± 2.85	30 µM	–	93.14 ± 0.45<br>[14.22]
T47D	60 µg/mL	40.64 ± 0.88	10 µM	20.88 ± 2.38	35.10 ±2.37
	90 µg/mL	44.05 ± 1.00	30 µM	22.02 ± 1.46	92.75 ± 0.99<br>[11.57]
MCF7	60 µg/mL	38.00 ± 1.81	10 µM	–	40.80 ± 2.39
	90 µg/mL	55.55 ± 2.70	30 µM	–	93.61 ± 0.77<br>[11.22]
HeLa	60 µg/mL	52.65 ± 2.67	10 µM	–	10.19 ± 1.44
	90 µg/mL	61.05 ± 2.41	30 µM	–	98.08 ± 0.24<br>[14.79]
A2780	60 µg/mL	54.88 ± 1.41	10 µM		56.59 ± 1.38
	90 µg/mL	63.20 ± 0.80	30 µM		99.27 ± 0.12<br>[0.688]
SiHa	60 µg/mL	46.90 ± 1.12	10 µM	–	15.80 ± 1.95
	90 µg/mL	48.40 ± 1.03	30 µM	17.39 ± 1.79	93.99 ± 0.47<br>[15.17]
C33A	60 µg/mL	60.09 ± 0.58	10 µM	–	69.34 ± 1.63
	90 µg/mL	63.69 ± 0.89	30 µM	–	97.87 ± 0.32<br>[0.845]
NIH/3T3	60 µg/mL	20.99 ± 1.69	10 µM	–	21.23 ± 1.33
	90 µg/mL	38.76 ± 0.57	30 µM	–	86.98 ± 1.52<br>[15.63]

[1]: Inhibition values less than 10% are considered negligible and not given numerically.

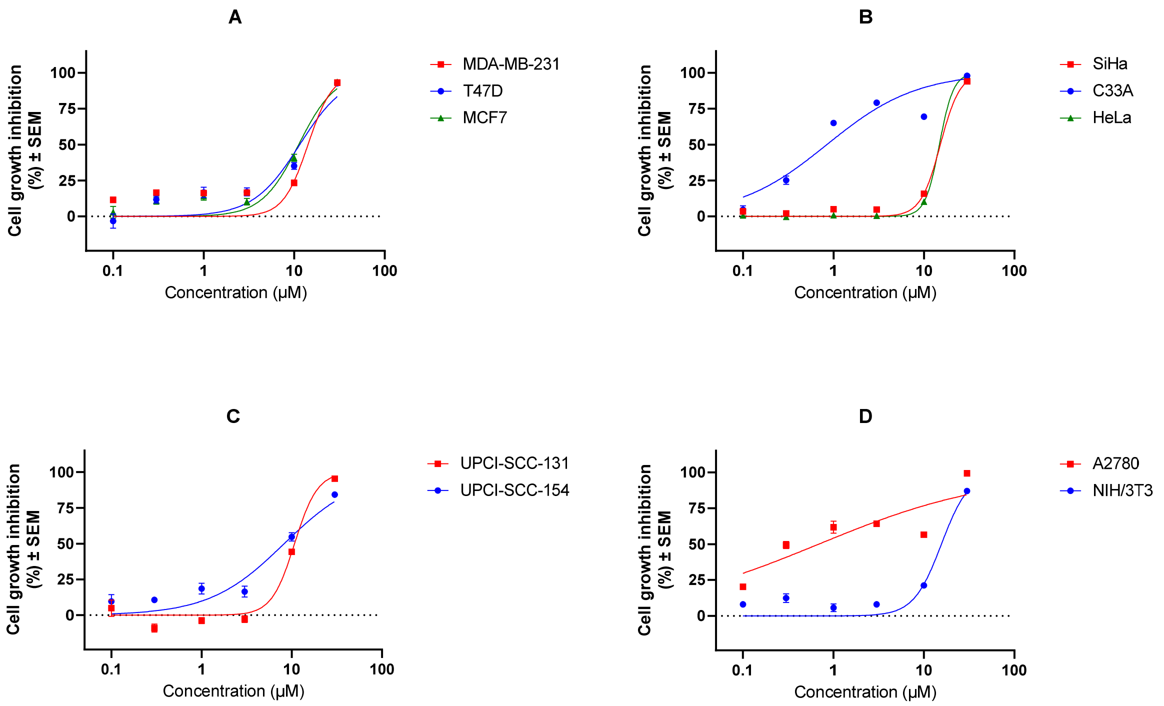

Figure S7. Antiproliferative effect of [6]-shogaol on cancer cell lines. **A)** Breast cancer cell lines. **B)** Cervical cancer cell lines. **C)** Oropharyngeal cancer cell lines. **D)** A2780 (ovarian cancer cell line) and NIH/3T3 (murine fibroblast cell line).

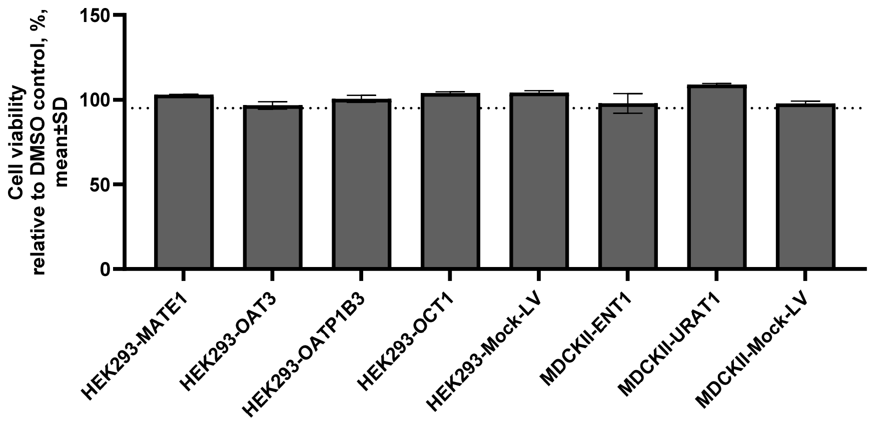

Figure S8. The acute effect of a 40-minute treatment with 30 µM [6]-shogaol on the viability of cell lines whose proliferation was negatively affected by a 72-hour treatment. Viability remained above 95% (dashed line) in all cell lines investigated.

References

1. Scalise, M.; Console, L.; Cosco, J.; Pochini, L.; Galluccio, M.; Indiveri, C. ASCT1 and ASCT2: Brother and Sister? *SLAS Discovery* 2021, *26*, 1148–1163, doi:10.1177/24725552211030288.

2. Boswell-Casteel, R.C.; Hays, F.A. Equilibrative Nucleoside Transporters—A Review. *Nucleosides Nucleotides Nucleic Acids* 2017, *36*, 7–30, doi:10.1080/15257770.2016.1210805.

3. Zhou, M.; Xia, L.; Wang, J. Metformin Transport by a Newly Cloned Proton-Stimulated Organic Cation Transporter (Plasma Membrane Monoamine Transporter) Expressed in Human Intestine. *Drug Metabolism and Disposition* 2007, *35*, 1956–1962, doi:10.1124/dmd.107.015495.

4. del Amo, E.M.; Urtti, A.; Yliperttula, M. Pharmacokinetic Role of L-Type Amino Acid Transporters LAT1 and LAT2. *European Journal of Pharmaceutical Sciences* 2008, *35*, 161–174, doi:10.1016/j.ejps.2008.06.015.

5. Chien, H.-C.; Colas, C.; Finke, K.; Springer, S.; Stoner, L.; Zur, A.A.; Venteicher, B.; Campbell, J.; Hall, C.; Flint, A.; et al. Reevaluating the Substrate Specificity of the L-Type Amino Acid Transporter (LAT1). *J Med Chem* 2018, *61*, 7358–7373, doi:10.1021/acs.jmedchem.8b01007.

6. Motohashi, H.; Inui, K. Multidrug and Toxin Extrusion Family SLC47: Physiological, Pharmacokinetic and Toxicokinetic Importance of MATE1 and MATE2-K. *Mol Aspects Med* 2013, *34*, 661–668, doi:10.1016/j.mam.2012.11.004.

7. Anwer, M.S.; Stieger, B. Sodium-Dependent Bile Salt Transporters of the SLC10A Transporter Family: More than Solute Transporters. *Pflugers Arch* 2014, *466*, 77–89, doi:10.1007/s00424-013-1367-0.

8. Li, T.-T.; An, J.-X.; Xu, J.-Y.; Tuo, B.-G. Overview of Organic Anion Transporters and Organic Anion Transporter Polypeptides and Their Roles in the Liver. *World J Clin Cases* 2019, *7*, 3915–3933, doi:10.12998/wjcc.v7.i23.3915.

9. Ye, J.; Liu, Q.; Wang, C.; Meng, Q.; Sun, H.; Peng, J.; Ma, X.; Liu, K. Benzylpenicillin Inhibits the Renal Excretion of Acyclovir by OAT1 and OAT3. *Pharmacological Reports* 2013, *65*, 505–512, doi:10.1016/S1734-1140(13)71026-0.

10. Samodelov, S.L.; Kullak-Ublick, G.A.; Gai, Z.; Visentin, M. Organic Cation Transporters in Human Physiology, Pharmacology, and Toxicology. *Int J Mol Sci* 2020, *21*, 7890, doi:10.3390/ijms21217890.

11. Abdul-Ghani, M.A.; DeFronzo, R.A. Dapagliflozin for the Treatment of Type 2 Diabetes. *Expert Opin Pharmacother* 2013, *14*, 1695–1703, doi:10.1517/14656566.2013.812632.

12. Ganapathy, V.; Smith, S.B.; Prasad, P.D. SLC19: The Folate/Thiamine Transporter Family. *Pflügers Archiv European Journal of Physiology* 2004, *447*, 641–646, doi:10.1007/s00424-003-1068-1.

13. Liang, X.; Chien, H.-C.; Yee, S.W.; Giacomini, M.M.; Chen, E.C.; Piao, M.; Hao, J.; Twelves, J.; Lepist, E.-I.; Ray, A.S.; et al. Metformin Is a Substrate and Inhibitor of the Human Thiamine Transporter, THTR-2 (SLC19A3). *Mol Pharm* 2015, *12*, 4301–4310, doi:10.1021/acs.molpharmaceut.5b00501.

14. Jamshidi, N.; Nigam, K.B.; Nigam, S.K. Loss of the Kidney Urate Transporter, Urat1, Leads to Disrupted Redox Homeostasis in Mice. *Antioxidants* 2023, *12*, 780, doi:10.3390/antiox12030780.

15. Mao, Q.; Unadkat, J.D. Role of the Breast Cancer Resistance Protein (BCRP/ABCG2) in Drug Transport—an Update. *AAPS J* 2015, *17*, 65–82, doi:10.1208/s12248-014-9668-6.

16. Kubitz, R.; Dröge, C.; Stindt, J.; Weissenberger, K.; Häussinger, D. The Bile Salt Export Pump (BSEP) in Health and Disease. *Clin Res Hepatol Gastroenterol* 2012, *36*, 536–553, doi:10.1016/j.clinre.2012.06.006.

17. Mollazadeh, S.; Sahebkar, A.; Hadizadeh, F.; Behravan, J.; Arabzadeh, S. Structural and Functional Aspects of P-Glycoprotein and Its Inhibitors. *Life Sci* 2018, *214*, 118–123, doi:10.1016/j.lfs.2018.10.048.

18. Toyoda, Y.; Hagiya, Y.; Adachi, T.; Hoshijima, K.; Kuo, M.T.; Ishikawa, T. MRP Class of Human ATP Binding Cassette (ABC) Transporters: Historical Background and New Research Directions. *Xenobiotica* 2008, *38*, 833–862, doi:10.1080/00498250701883514.

19. Kiser, J.; Carten, M.; Aquilante, C.; Anderson, P.; Wolfe, P.; King, T.; Delahunty, T.; Bushman, L.; Fletcher, C. The Effect of Lopinavir/Ritonavir on the Renal Clearance of Tenofovir in HIV-Infected Patients. *Clin Pharmacol Ther* 2008, *83*, 265–272, doi:10.1038/sj.clpt.6100269.

20. Reid, G.; Wielinga, P.; Zelcer, N.; de Haas, M.; van Deemter, L.; Wijnholds, J.; Balzarini, J.; Borst, P. Characterization of the Transport of Nucleoside Analog Drugs by the Human Multidrug Resistance Proteins MRP4 and MRP5. *Mol Pharmacol* 2003, *63*, 1094–1103, doi:10.1124/mol.63.5.1094.
